# Confined Synthesis of Amorphous Al_2_O_3_ Framework Nanocomposites Based on the Oxygen‐Potential Diagram as Sulfur Hosts for Catalytic Conversion

**DOI:** 10.1002/advs.202302215

**Published:** 2023-06-19

**Authors:** Pengbiao Geng, Yuxing Lin, Meng Du, Chunsheng Wu, Tianxing Luo, Yi Peng, Lei Wang, Xinyuan Jiang, Shuli Wang, Xiuyun Zhang, Lubin Ni, Shuangqiang Chen, Mohsen Shakouri, Huan Pang

**Affiliations:** ^1^ School of Chemistry and Chemical Engineering Yangzhou University Yangzhou Jiangsu 225009 P. R. China; ^2^ College of Physics Science and Technology Yangzhou University Yangzhou Jiangsu 225009 P. R. China; ^3^ Department of Chemical Engineering School of Environmental and Chemical Engineering Shanghai University Shanghai 200444 P. R. China; ^4^ Canadian Light Source Inc. University of Saskatchewan Saskatoon S7N 2V3 Canada

**Keywords:** amorphous aluminum oxide, catalysis, confined synthesis, Ni nanocrystals, oxygen‐potential diagram

## Abstract

Sulfur cathodes in Li–S batteries suffer significant volumetric expansion and lack of catalytic activity for polysulfide conversion. In this study, a confined self‐reduction synthetic route is developed for preparing nanocomposites using diverse metal ions (Mn^2+^, Co^2+^, Ni^2+^, and Zn^2+^)‐introduced Al‐MIL‐96 as precursors. The Ni^2+^‐introduced Al‐MIL‐96‐derived nanocomposite contains a “hardness unit”, amorphous aluminum oxide framework, to restrain the volumetric expansion, and a “softness unit”, Ni nanocrystals, to improve the catalytic activity. The oxygen‐potential diagram theoretically explains why Ni^2+^ is preferentially reduced. Postmortem microstructure characterization confirms the suppressive volume expansion. The in situ ultraviolet–visible measurements are performed to probe the catalytic activity of polysulfide conversion. This study provides a new perspective for designing nanocomposites with “hardness units” and “softness units” as sulfur or other catalyst hosts.

## Graphical abstract

This study designs a confined self‐reduction route for preparing nanocomposites using diverse metal ions‐introduced Al‐MIL‐96 as precursors. The obtained nanocomposite containing “hardness unit”, amorphous Al_2_O_3_ framework, can restrain the volumetric expansion and “softness unit”, Ni nanocrystals, can improve the catalytic activity for polysulfide conversion. This work provides a new strategy for designing sulfur or other catalyst hosts.

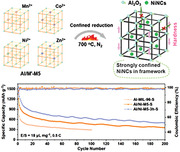

## 1 Introduction

To meet the ever‐growing demand for energy storage devices, Li–S batteries have been proposed as one of the most promising substitutes for Li‐ion batteries because of the natural abundance and high theoretical specific capacity of sulfur.^[^ ^1^, ^2^, ^3^ ^]^ However, sulfur cathodes suffer significant volumetric expansion and the shuttle effect of lithium polysulfides (LPS, chemical formula: Li_2_S*_n_*, 4 ≤ *n* ≤ 8) during cycling, which results in poor cyclic performance.^[^ ^4^, ^5^, ^6^ ^]^ Different host materials have been proposed to solve these problems, including carbonaceous materials and transition metal oxides,^[^ ^7^, ^8^ ^]^ but nonpolar carbonaceous materials exhibit low specific capacity owing to the weak adsorption ability for LPS;^[^ ^9^, ^10^ ^]^ and most transition metal oxides exhibit dilapidated frameworks because of the reaction with LPS.^[^ ^11^ ^]^ Thus, a host material with strong adsorption ability for LPS and chemical inertness is required, which should also accompany a stable framework for loading sulfur.

Metal–organic frameworks (MOFs) have garnered considerable attention as sulfur host materials owing to their abundant free space.^[^ ^12^, ^13^ ^]^ However, when MOFs are directly used as sulfur hosts, they exhibit a lack of chemical adsorption to LPS. Some researchers have considered introducing a second metal ion to improve the chemical adsorption between MOFs and LPS.^[^ ^14^, ^15^ ^]^ Furthermore, using MOFs as precursors in the preparation of MOF‐derived metal oxide/carbon nanocomposites is an effective method for improving the electron transfer rate.^[^ ^16^, ^17^, ^18^ ^]^ For example, Lee and co‐workers obtained a Co/Co_3_O_4_ nanoparticles‐embedded hollow carbon composite as sulfur host using a core–shell structured bimetallic Zn/Co‐MOFs@ZnO as template.^[^ ^19^ ^]^ But, the intrinsic catalytic activity for LPS conversion needs to be improved. Recent studies have demonstrated that metal nanocrystals can serve as efficient catalysts to accelerate LPS conversion and improve the reaction kinetics.^[^ ^20^, ^21^, ^22^ ^]^ The existing MOF‐derived metal nanocrystal composites always have a destroyed, relatively closed sulfur‐loading structure, and the synthetic process is relatively complicated and carries a risk of explosion.^[^ ^23^, ^24^ ^]^ Therefore, a synthesis method for preparing host materials containing effective nanocatalysts needs to be explored.

As a type of MOFs,^[^ ^25^, ^26^ ^]^ Al‐MIL‐96 with a 3D structure consisting of Al octahedral units connected by 1,3,5‐benzenetricarboxylate ligands exhibits a tunable pore structure and chemical stability.^[^ ^27^, ^28^ ^]^ In comparison with other MOFs,^[^ ^29^, ^30^, ^31^ ^]^ Al‐MIL‐96 is a polyhedral MOF that contains three different types of cavities (spherical cages, elongated cavities, and narrow cages), which give it a flexible structure for introducing other metal ions.^[^ ^32^, ^33^ ^]^ In the coordination units, Al atoms are coordinated with O atoms.^[^ ^34^ ^]^ This is also beneficial for the introduction of other metal ions owing to the affinity between metal ions and O atoms. In addition, Al‐MIL‐96 contains a large number of hydroxyl groups that can effectively confine metal ions. The rigidity of Al_2_O_3_, which is generated after pyrolysis of Al‐MIL‐96 at a high temperature, can maintain the internal regular framework, and the obtained metal nanocrystals are confined within the framework. Therefore, Al‐MIL‐96 is a promising parent material for introducing other metal ions.^[^ ^35^ ^]^

In this study, a one‐pot cosolvent method was employed to introduce Mn^2+^, Co^2+^, Ni^2+^, and Zn^2+^. After the introduction of these metal ions, uniform sizes were obtained, and the Ni^2+^‐introduced Al‐MIL‐96 exposed a specific crystal plane (100). The coordination of the second metal ions with the O atoms was revealed through X‐ray absorption fine structure spectroscopy. Moreover, to improve the electron transfer rate and catalytic activity for LPS conversion, we developed a confined self‐reduction synthetic route for preparing nanocomposites using the as‐obtained diverse metal ion‐introduced Al‐MIL‐96 as precursors. Interestingly, only Ni^2+^‐introduced Al‐MIL‐96 obtained Ni nanocrystals (NiNCs), which endowed the nanocomposite with effective chemical adsorption and catalytic activity against LPS. In addition, we analyzed why Ni^2+^ can be preferentially reduced to NiNCs. According to the oxygen‐potential diagram, it is theoretically explained that the reduction of NiNCs is reasonable, however, Al can only transform into Al_2_O_3_. More importantly, the confined synthetic route can keep the NiNCs in the interior of the nanocomposites, which exhibits more efficient catalysis to accelerate LPS conversion. To adequately demonstrate the chemical adsorption and catalytic activity of NiNCs, we further removed the NiNCs from the nanocomposite, and the Li–S battery test demonstrated that the specific capacity decreased sharply.

The “hardness unit”, Al_2_O_3_ framework, can support the skeleton of nanocomposite to store sulfur for suppressing the shuttle effect. Moreover, it exhibits chemical reaction inertness with LPS and high stability over a wide voltage range. The “softness unit”, NiNCs, can catalyze the conversion of long‐chain LPS. Hence, the as‐prepared amorphous aluminum oxide framework nanocomposite acting as a sulfur host exhibited the synchronous characteristics of a stable framework and catalytic activity; hence, excellent Li–S battery performance (initial specific capacity of 1233.8 mAh g^−1^ at 0.5 C) was achieved. The scanning electron microscopy (SEM) images of the cycled electrode indicated structural hardness, and in situ ultraviolet–visible (UV/vis) measurements were performed to probe the catalytic activity in LPS conversion. Finally, this synthetic route of nanocomposite with “hardness unit” and “softness unit” provides a new perspective to design sulfur hosts or other catalyst hosts.

## 2 Results and Discussion

**Figure 1:**
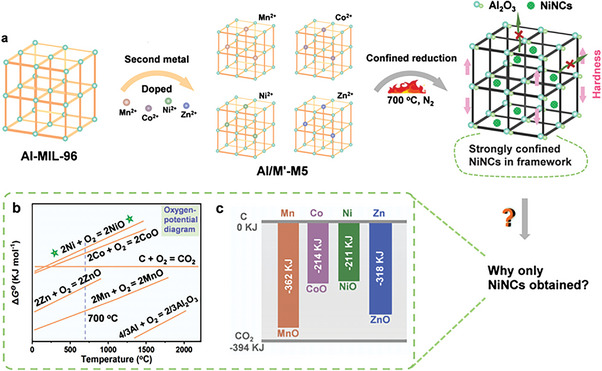
a) Schematic of Al/M′‐M*x* (M′ denotes the Mn^2+^, Co^2+^, Ni^2+^, and Zn^2+^; M denotes Al‐MIL‐96; *x* = 1, 5, and 10 indicates the molar ratio of Al/M′ = 10:1, 5:1, and 1:1), and confined synthesis of framework nanocomposites. b) Oxygen‐potential diagram of the covered metal oxides. c) Δ_f_ *G* ^Θ^_m_ of different metal oxides.

**Figure 2:**
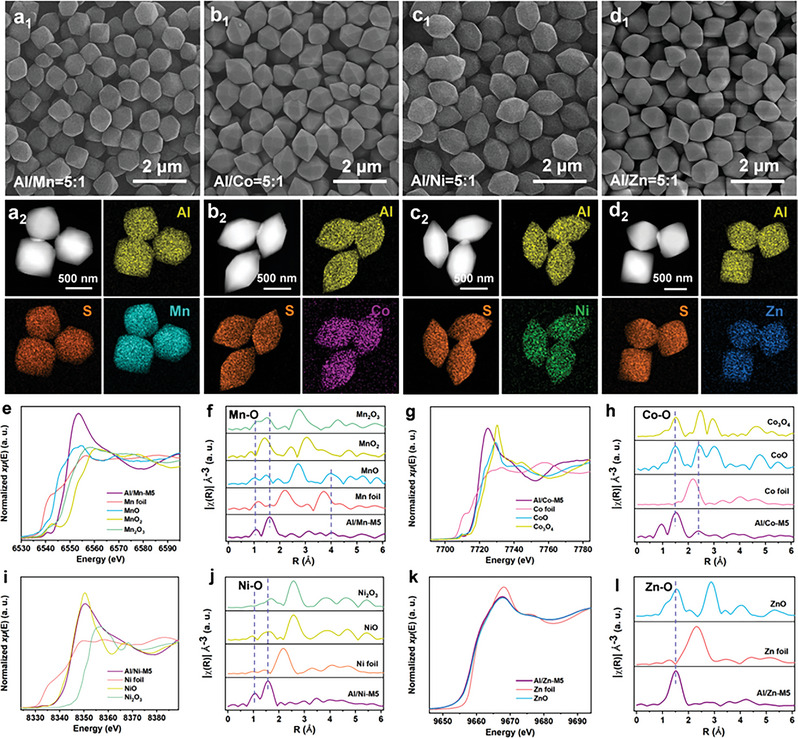
a_1_–d_1_) SEM images of Al/M′‐M5. a_2_–d_2_) Corresponding elemental mapping images. e) Mn K‐edge XANES spectra and f) EXAFS spectra. g) Co K‐edge XANES spectra and h) EXAFS spectra. i) Ni K‐edge XANES spectra and j) EXAFS spectra. k) Zn K‐edge XANES spectra and l) EXAFS spectra.

Diverse metal ions were introduced into Al‐MIL‐96 using a cosolvent method, and the confined self‐reduction method was used to obtain a NiNC‐containing framework nanocomposite. The synthetic route is illustrated in **Figure** **1**a and the detailed process is presented in the Supporting Information. The obtained diverse metal ion‐introduced Al‐MIL‐96 is referred to as Al/M′‐M*x*. When the ratio is Al/M′ = 10:1, the obtained Al/M′‐M1 can maintain the initial hexagonal bipyramidal shape of Al‐MIL‐96 (Figure S1a,b and Figure S2a,d, Supporting Information). As the molar ratio of Al/M′ increased to 5:1, a new crystal plane (100) was exposed in Al/Co‐M5 and Al/Ni‐M5 (**Figure 2** b_1_,c_1_). The SEM images of Al/M′‐M1 and Al/M′‐M5 show that all these samples have uniform sizes (Figure 2a_1_–d_1_). The particle sizes of Al/M′‐M10 exhibited a sudden increase when the Al/M′ ratio was 1:1 (Figure S2e–h, Supporting Information). Meanwhile, Al/Ni‐M10 and Al/Zn‐M10 generated hydroxides or oxides owing to the excess metal cations. The inductively coupled plasma optical emission spectrometry analysis summarized in Table S1 of the Supporting Information for Al/M′‐M*x* as a representative indicates the actual molar ratio of Al/M′.

The X‐ray diffraction (XRD) patterns of Al‐MIL‐96 and Al/M′‐M*x* matched well with those of simulated Al‐MIL‐96 owing to their similar unit cells and crystal lattices (Figure S1c and Figure S3, Supporting Information). Here, we selected Al/M′‐M5 with uniform size as the sulfur host to study the effect of different metal ion introductions on the shuttle effect. To prepare the cathode materials, sulfur was impregnated into the pores of Al/M′‐M5 via a melt‐diffusion process at 155 °C, and the products were referred to as Al/M′‐M5‐S. The SEM images of the sulfur‐loaded cathode materials are illustrated in Figure S4a–d of the Supporting Information, which indicate that Al/M′‐M5 maintained their initial shapes owing to their thermostability. The XRD patterns of sulfur‐loaded Al/M′‐M5‐S exhibited distinct S_8_ signals (Figure S4e–h, Supporting Information). Scanning transmission electron microscopy and energy dispersive X‐ray (EDX) mapping of Al/M′‐M5‐S (Figure 2a_2_–d_2_) demonstrated the uniform distribution of the second metal and sulfur. The N_2_ adsorption isotherms at 77 K (Figure S5, Supporting Information) suggest that the obtained Al/M′‐M5 has porous features similar to those of Al‐MIL‐96, as reported in our previous study.^[^ ^27^ ^]^ As expected, all the sulfur‐loaded samples exhibited a significant loss in N_2_ adsorption capacity owing to the pores being occupied by sulfur. Pore volume data are important indicators for determining the sulfur loading content. According to the pore volume data and density of sublimed sulfur, the theoretical amount of sulfur loaded was calculated, and the results are summarized in Table S2 of the Supporting Information. Considering these results, the sulfur content was controlled at a mass percentage of 60% by governing the feeding ratio, and the precise sulfur mass percent was tested using the thermogravimetric analysis (TGA) profiles (Figure S6, Supporting Information) and elemental analysis data listed in Table S3 of the Supporting Information. Raman spectroscopy was used to confirm the presence of sulfur. The peaks at ≈1006 and 1460 cm^−1^ were assigned to C—O and C=C bonds (Figure S7a–c, Supporting Information), respectively. After the sulfur loading process, the bands at ≈152, 219, and 469 cm^−1^ were assigned to the S_8_ molecule (Figure S7d, Supporting Information).

To explore the surface electronic structure and chemical bonding of Al/M′‐M5 and Al/M′‐M5‐S, X‐ray photoelectron spectroscopy (XPS) analysis was performed (Figure S8, Supporting Information). The high‐resolution Mn, Co, Ni, and Zn 2p XPS spectra show that the cations mainly exist in the chemical valences of +2 and +3. After the sulfur loading process, the 2p XPS spectra exhibits Mn—S, Co—S, and Ni—S bonds.^[^ ^36^ ^]^ For Al/Zn‐M5, owing to the low interaction between Zn and sulfur, the high‐resolution Zn 2p XPS spectra did not exhibit the Zn–S signal. To further illustrate the interaction between Al/M′‐M5 and sulfur, high‐resolution S 2p XPS spectra of Al/M′‐M5‐S were analyzed. A broad peak located at ≈168.6 eV indicates the formation of sulfate species. Moreover, it can be observed that the S 2p spectra of Al/Ni‐M5‐S exhibit a clear S–Ni signal (Figure S9, Supporting Information). This confirms the strong chemical bonding between sulfur and Al/Ni‐M5.

In addition to the XPS analysis, X‐ray absorption fine structure (XAFS) characterization was also conducted to analyze the structure of Al/M′‐M5. The X‐ray absorption near‐edge structure (XANES) spectra at the Mn K‐edge for Al/Mn‐M5 with Mn foil and MnO*_x_* as references are shown in Figure 2e. The adsorption edge of Al/Mn‐M5 was compared to that of MnO and Mn_2_O_3_, confirming the existence of +2 and +3 chemical valences. The peaks at 1.07 and 1.60 Å in extended XAFS (EXAFS) of the Mn K‐edge are close to that of Mn_2_O_3_, and the peak at 4.01 Å is assigned to MnO (Figure 2f). The Co K‐edge XANES spectra of Al/Co‐M5 (Figure 2g) show that the valence of Co is close to +2 and it exhibits a weak shift to +3. The EXAFS spectra of the Co K‐edge exhibit two peaks at 1.50 and 2.37 Å, which is close to the CoO and Co_3_O_4_ bonds, respectively (Figure 2h). The Ni K‐edge XANES spectra of Al/Ni‐M5 are shown in Figure 2i and characterize the +2 and +3 chemical valences. The EXAFS spectra at the Ni K‐edge exhibit two peaks at 1.05 and 1.58 Å, which are assigned to NiO (Figure 2j). The Zn K‐edge XANES spectra of Al/Zn‐M5 with Zn foil and ZnO as references are shown in Figure 2k. The adsorption edges of Al/Zn‐M5 confirm the existence of a chemical valence of +2. The peaks at 1.51 Å in the EXAFS spectrum of the Zn K‐edge are the same as those of ZnO (Figure 2l), indicating the coordination of Zn–O.

To obtain a mechanistic understanding of the improved LPS adsorption ability for LPS and Al/M′‐M5, density functional theory calculations were performed. Figure S10 of the Supporting Information shows the optimized adsorption configurations of the Al‐MIL‐96 (002) plane and second metal‐introduced configurations with S_8_ and Li_2_S_4_. The calculated binding energies of the (002) plane of Al‐MIL‐96 and Al/M′‐M5 to S_8_ molecules were −0.09, −0.56, −0.85, −1.46, and −0.11 eV, and the binding energies to Li_2_S_4_ molecules were −0.19, −0.78, −1.05, −2.56, and −1.31 eV. The calculated results revealed the larger adsorption ability of the Ni‐introduced configuration.^[^ ^37^ ^]^ To test the LPS adsorption ability on a macroscopic scale, Li_2_S_4_ permeation measurements of Al‐MIL‐96 and Al/M′‐M5 were conducted (Figure S11, Supporting Information). The brown Li_2_S_4_‐containing solution became transparent owing to chemical adsorption. The color change of the Li_2_S_4_ solution indicated that Al/Ni‐M5 achieved rapid color fading. We speculate that the second metal ions in the crystal orientation of the MOF particles play a vital role in facilitating LPS encapsulation. Accordingly, the Li_2_S_4_ concentration changes in the supernatants were monitored using UV/vis spectroscopy. The absorption intensity of the Al/Ni‐M5 supernatant at 420 nm after 3 h was weaker than those of the other samples (Figure S12, Supporting Information). This was consistent with the subsequent macroscopic LPS adsorption results.

Cyclic voltammetry (CV) was performed to investigate the redox reactions between S_8_ and LPS. Figure S13 of the Supporting Information shows a comparison of the CV curves during the third cycle of Al/M′‐M5‐S at a scan rate of 0.1 mV s^−1^. The CV curves showed two reduction peaks. A minor peak at ≈2.30 V is attributed to the conversion of S_8_ to LPS, which is a solid–liquid‐phase reaction with relatively rapid kinetics. A major peak at ≈2.05 V corresponds to the liquid–solid‐phase conversion from Li_2_S_4_ to Li_2_S_2_/Li_2_S.^[^ ^38^ ^]^ Owing to its high energy barrier and slow kinetics, the later process is usually the rate‐determining step. The Al/Ni‐M5‐S electrode exhibited a lower anodic potential and higher cathodic potential than the others, indicating that Al/Ni‐M5‐S can significantly reduce electrode polarization and improve LPS redox kinetics. To study their influence on electrochemical stability, the long‐term cyclic performance at 0.5 C using Al/M′‐M5 as the host material was tested. The initial specific capacities of the assembled cells with Al‐MIL‐96‐S, Al/Mn‐M5‐S, Al/Co‐M5‐ S, Al/Ni‐M5‐S, and Al/Zn‐M5‐S (Figure S14, Supporting Information) were 895.1, 1136.2, 1206.7, 1216.8, and 1111.0 mAh g^−1^, respectively. After 100 cycles, the specific capacities were 290.6, 373.0, 414.0, 456.6, and 319.3 mAh g^−1^, respectively. Owing to the low LPS adsorption ability of Al‐MIL‐96, the specific capacity of Al‐MIL‐96‐S rapidly decreased. The replacement of higher‐valent Al^3+^ by lower‐valent metal ions induced an internal dipole, which contributed to the interaction between the framework and LPS.

**Figure 3:**
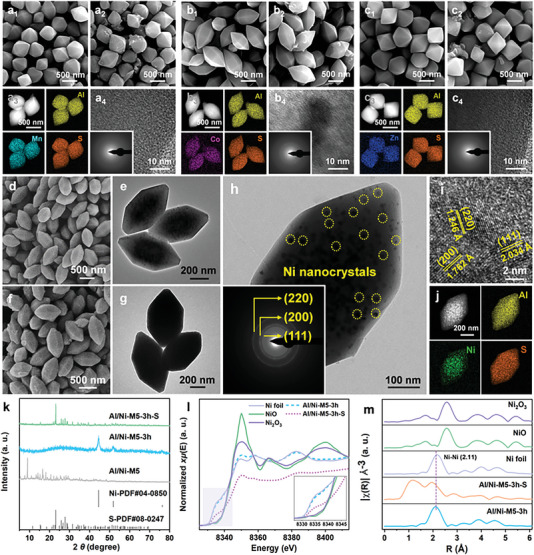
SEM images of a_1_) Al/Mn‐M5‐3 h and a_2_) Al/Mn‐M5‐3h‐S. a_3_) Elemental mapping images and a_4_) HRTEM and SAED pattern of Al/Mn‐M5‐3h‐S. SEM images of b_1_) Al/Co‐M5‐3 h and b_2_) Al/Co‐M5‐3h‐S. b_3_) Elemental mapping images and b_4_) HRTEM and SAED pattern of Al/Co‐M5‐3h‐S. SEM images of c_1_) Al/Zn‐M5‐3 h and c_2_) Al/Zn‐M5‐3h‐S. c_3_) Elemental mapping images and c_4_) HRTEM and SAED pattern of Al/Zn‐M5‐3h‐S. SEM images of d) Al/Ni‐M5‐3 h and f) Al/Ni‐M5‐3h‐S. TEM images of e) Al/Ni‐M5‐3 h and g) Al/Ni‐M5‐3h‐S. h) Magnified TEM image and SAED pattern, i) HRTEM image, and j) elemental mapping images of Al/Ni‐M5‐3h‐S. k) XRD patterns of Al/Ni‐M5, Al/Ni‐M5‐3 h, Al/Ni‐M5‐3h‐S, and simulated S, Ni. l) Ni K‐edge XANES spectra and m) EXAFS spectra of Al/Ni‐M5‐3 h and Al/Ni‐M5‐3h‐S.

Before the confined synthesis process, the thermal stability of Al/M′‐M5 was tested using the TGA method. Because they were synthesized using a solvothermal method in an organic solvent and deionized water, some solvent molecules were retained inside the crystals or adsorbed onto the surface. These solvents were released during the initial heating process, resulting in weight loss (Figure S15, Supporting Information). More specifically, Al/M′‐M5 reached a weight‐loss platform after ≈700 °C. To obtain the MOF‐derived metal nanocrystal, the dried Al/M′‐M5 was heated to 700 °C at a heating rate of 3 °C min^−1^ in a N_2_ atmosphere and maintained for 3 h. MOF‐derived samples were named Al/M′‐M5‐3 h. **Figure 3** a_1_–c_1_,d shows the typical SEM images of Al/M′‐M5‐3 h. The particle size became smaller than that of Al/M′‐M5 after pyrolysis. The transmission electron microscopy (TEM) images of Al/M′‐M5‐3 h illustrated in Figure 3e and Figure S16a–h (Supporting Information) show that only Al/Ni‐M5‐3 h exhibited distinct metal nanocrystals after calcination. However, the contrast‐adjusted TEM image of Al/Co‐M5‐3 h illustrated in Figure S16i of the Supporting Information also shows bright spots in the center of the particles. The XRD patterns shown in Figure S17 of the Supporting Information also demonstrate the presence of metallic Ni in Al/Ni‐M5‐3 h, whereas Al/Mn‐M5‐3 h and Al/Zn‐M5‐3 h only exhibit amorphous carbon signals. Interestingly, Al/Co‐M5‐3 h exhibits a weak Co metal signal, which is consistent with the TEM result illustrated in Figure S16i of the Supporting Information. These results are guided by the following oxygen potential diagram.

The sulfur loading process was the same as the melt‐diffusion process described above. The XRD patterns of the obtained sulfur‐loaded samples (named Al/M′‐M5‐3h‐S) exhibited distinct sulfur signals (Figure S18, Supporting Information). The SEM images of Al/M′‐M5‐3h‐S illustrated in Figure 3a_2_–c_2_,f shows no sulfur powder leaving the host materials. Owing to sulfur impregnation, the filled inner space of Al/Ni‐M5‐3 h observed in the TEM image of Al/Ni‐M5‐3h‐S (Figure 3g) indicates that most of the sulfur was confined in the host material. The EDX mapping of Al/M′‐M5‐3h‐S (Figure 3a_3_–c_3_,j) demonstrates the uniform distribution of Al, the second metal, and S in Al/M′‐M5‐3h‐S. The magnified TEM image and SAED pattern illustrated in Figure 3h demonstrate the uniform distribution of NiNCs and the clear diffraction rings of metallic Ni, respectively. The high‐resolution TEM (HRTEM) image illustrated in Figure 3i shows the lattice fringes of the NiNCs with interplanar spacings of 1.246, 1.762, and 2.034 Å, which correspond to the (220), (200), and (111) planes of metallic Ni, respectively. The HRTEM images and SAED patterns of Al/Mn‐M5‐3h‐S and Al/Zn‐M5‐3h‐S (Figure 3a_4_,c_4_) demonstrate their amorphous characteristics. However, the HRTEM image and SAED pattern of Al/Co‐M5‐3h‐S (Figure 3b_4_) indicate that a small quantity of Co nanocrystals is confined to the interior of the amorphous Al_2_O_3_/carbon framework nanocomposite. The oxygen‐potential diagram illustrated in Figure 1b explains why Al/Ni‐M5 can easily be reduced to NiNCs among the Al/M′‐M5 samples by the carbon obtained in the pyrolysis process. The ordinate represents the Gibbs free energy of different metal oxides, which is more positive, and the corresponding metal oxide is easier to reduce because the metal particles are formed by carbon. According to the Δ_f_ *G* ^Θ^_m_ values of the covered metal oxides illustrated in Figure 1c, NiO exhibits the lowest Gibbs free energy, indicating the highest reducibility. Hence, among the Al/M′‐M5 samples, Al/Ni‐M5 was first reduced to metal nanocrystals.

The XRD pattern of Al/Ni‐M5‐3 h (Figure 3k) at 44.5°, 51.8°, and 76.4° correlate well with the (111), (200), and (220) planes of metallic Ni, respectively. After sulfur loading, Al/Ni‐M5‐3h‐S exhibits very sharp peaks of sulfur, which also confirms the loading of sulfur within Al/Ni‐M5‐3 h. In comparison with Al/Ni‐M5, pyrolysis will result in a slight decline in the N_2_ adsorption quantity (Figure S19, Supporting Information). After sulfur loading, this phenomenon continued. The Ni K‐edge XANES spectra illustrated in Figure 3l shows that the absorption edges of Al/Ni‐M5‐3 h were placed near the Ni foil. Figure 3m shows the Fourier‐transformed EXAFS spectra, which were used to explore the chemical environment of the Ni atoms in the host material. In comparison with the R‐space of Ni foil, NiO, and Ni_2_O_3_, the peaks at 2.11 Å were observed in Al/Ni‐M5‐3h‐S, which was attributed to the NiNCs. The absence of the Ni—Ni bond confirmed the presence of metallic Ni. A comparison of the XPS spectra before and after pyrolysis of Al/M′‐M5 illustrated in Figure S20 of the Supporting Information clearly demonstrates the presence of metallic Co and Ni.

To study the influence of different pyrolysis times on the NiNC content, we further selected pyrolysis times of 1 and 5 h, named Al/Ni‐M5‐1 h and Al/Ni‐M5‐5 h, respectively. The SEM images of Al/Ni‐M5‐1 h and Al/Ni‐M5‐5 h shown in Figure S21a,b of the Supporting Information, show the thermostability of Al/Ni‐M5. The corresponding TEM images illustrated in Figure S21c,d of the Supporting Information indicate that the NiNC content increased as the pyrolysis time increased. The HRTEM images, SAED patterns (Figure S21e,f, Supporting Information), and XRD patterns (Figure S22, Supporting Information) demonstrate the presence of NiNCs. Figure S23 of the Supporting Information shows the electron paramagnetic resonance spectra of the samples with different pyrolysis times at room temperature. In comparison with the parent Al/Ni‐M5, the Al/Ni‐M5‐1 h, ‐3 h, and ‐5 h samples exhibited strong signals at Gs values of 2411 and 3663, indicating the existence of metallic Ni. With the increase in heating time, the content of generated NiNCs increased, and the signal became increasingly clear. The two strong signals of Al/Ni‐M5‐3h‐S shifted to 277 and 225 Gs, owing to sulfur impregnation. After pyrolysis, the D and G bands in the Raman test emerged, and the ratio of *I*_D_/*I*_G_ increased with increasing pyrolysis time (Figure S24, Supporting Information).

The Al 2p XPS spectra of Al/Ni‐M5, Al/Ni‐M5‐1 h, Al/Ni‐M5‐3 h, Al/Ni‐M5‐5 h, and Al/Ni‐M5‐3h‐S (Figure S25, Supporting Information) exhibit a strong peak at 74.6 eV assigned to the Al—O bond of Al_2_O_3_, which is often used to inhibit surface degradation and counteract mechanical stress.^[^ ^39^ ^]^ With the increase of NiNC contents, a negative shift of the Al 2p peaks was observed. For the amorphous Al_2_O_3_ in Al/Ni‐M5‐derived samples, the binding energies of Al 2p shifted to lower values compared with those of Al/Ni‐M5 because of the lattice distortion of the amorphous Al_2_O_3_, shortening the interatomic distance and making it easier for electrons to hop over both Al and O elements.^[^ ^40^ ^]^ After sulfur loading, the shift reversed. The Ni 2p spectra of the Al/Ni‐ M5‐derived samples (Figure S26, Supporting Information) exhibited a metallic Ni signal owing to the obtained NiNCs.

**Figure 4:**
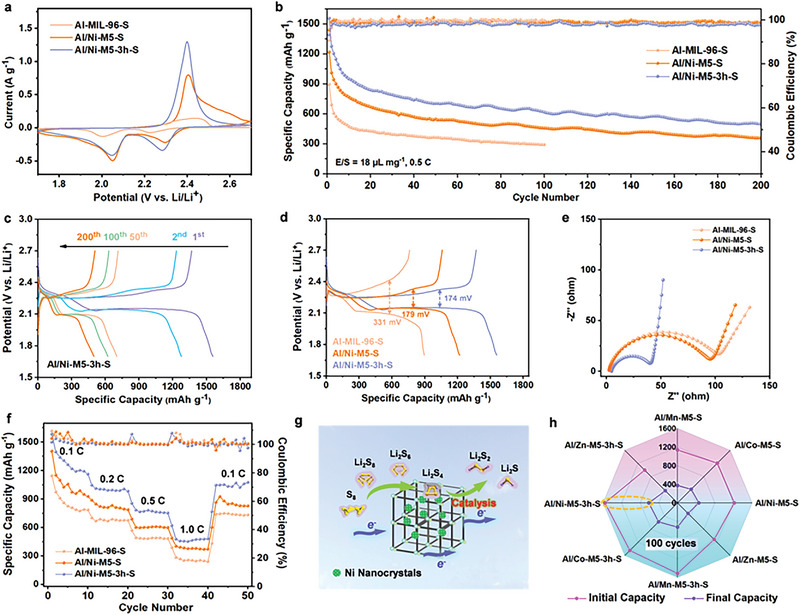
a) CV curves during third cycle of Al‐MIL‐96‐S, Al/Ni‐M5‐S, and Al/Ni‐M5‐3h‐S at 0.1 mV s^−1^. b) Cyclic performance of Al‐MIL‐96‐S, Al/Ni‐M5‐S, and Al/Ni‐M5‐3h‐S. c) GCD profiles of Al/Ni‐M5‐3h‐S. d) GCD profiles of Al‐MIL‐96‐S, Al/Ni‐M5‐S, and Al/Ni‐M5‐3h‐S. e) EIS test of Al‐MIL‐96‐S, Al/Ni‐M5‐S, and Al/Ni‐M5‐3h‐S before cyclic GCD process. f) Rate performance of Al‐MIL‐96, Al/Ni‐M5‐S, and Al/Ni‐M5‐3h‐S. g) Schematic illustration of catalytic conversion from LPS to Li_2_S_2_/Li_2_S by NiNCs in Al/Ni‐M5‐3 h. h) Radar map of the initial and final capacities of all electrode materials at 0.5 C.

The electrochemical performance of the Li–S batteries assembled with the Al/M′‐M5‐3 h nanocomposites as host materials was systematically investigated based on coin cells. The TGA curves (Figure S27, Supporting Information) and elemental analysis results (Table S4, Supporting Information) were used to obtain an accurate sulfur content. First, CV curves were obtained to analyze the reversibility of the electrode reaction. The compared CV curves illustrated in **Figure** **4**a show that minor cathodic peaks of Al/Ni‐M5‐S and Al/Ni‐M5‐3h‐S shift from 2.30 to 2.28 V, and the peak current of Al/Ni‐M5‐3h‐S increases.^[^ ^41^ ^]^ This phenomenon indicates the catalytic activity of the “softness unit”, NiNCs, in LPS conversion. The CV curves of Al/Mn‐M5‐3h‐S, Al/Co‐M5‐3h‐S, and Al/Zn‐M5‐3h‐S are shown in Figure S28 of the Supporting Information. The comparison of the long‐term cyclic performance of Al/M′‐M5‐3h‐S is illustrated in Figure 4b and Figure S29a (Supporting Information); the Al/Ni‐M5‐3h‐S electrode exhibits an improved cyclic stability, achieving a specific capacity of 496 mAh g^−1^ after 200 cycles at 0.5 C. Furthermore, the MOF‐derived carbon skeleton provides an electron conductive medium for improving the coulombic efficiency that can be maintained at ≈95%. The galvanostatic charge–discharge (GCD) profiles of Al/Ni‐M5‐3h‐S (Figure 4c) demonstrate that the two discharge plateaus can be maintained as the GCD process proceeds. When comparing the potential voltage gaps, the Al/Ni‐M5‐3h‐S electrode was the smaller (174 mV) than those of the Al‐MIL‐96‐S (331 mV) and Al/Ni‐M5‐S (179 mV) during the initial cycles (Figure 4d). This indicates that the polarization of the Al/Ni‐M5‐3h‐S cathode was lower than those of the Al‐MIL‐96‐S and Al/Ni‐M5‐S cathodes. Moreover, the Al/Ni‐M5‐3h‐S electrode with a high sulfur mass loading of ≈3.6 mg cm^−2^ can still display a reversible capacity of 415.2 mAh g^−1^ after 200 cycles at 0.5 C in Figure S29b of the Supporting Information. These results were ascribed to the efficient redox reaction kinetics of the carbon skeleton and NiNCs.

Electrochemical impedance spectroscopy was performed to investigate the kinetics behavior of the introduction of NiNCs. The semicircle in the high‐frequency region corresponds to the charge‐transfer resistance. The Nyquist plots illustrated in Figure 4e show that the charge transfer resistance of the Al/Ni‐M5‐3h‐S electrode is the lowest, which is attributed to the generated Warburg tail for the Al/Ni‐M5‐3h‐S electrode demonstrates a fast Li^+^ diffusion process owing to the porous framework.^[^ ^42^, ^43^ ^]^ The carbon skeleton. Furthermore, the steeper slope of the linear Nyquist plots of Al/Mn‐M5‐3h‐S, Al/Co‐M5‐3h‐S, and Al/Zn‐M5‐3h‐S illustrated in Figure S30 of the Supporting Information shows that Al/Ni‐M5‐5h‐S exhibits the smallest charge transfer resistance. This was ascribed to the inherent electrical conductivity determined by the carbon skeleton and NiNCs. In addition, the rate performances of Al‐MIL‐96‐S, Al/Ni‐M5‐S, and Al/Ni‐M5‐3h‐S were evaluated at 0.1–1.0 C (Figure 4f). The Al/Ni‐M5‐3h‐S electrode exhibited specific capacities of 1584.8, 1046.9, 855.8, and, 621.3 mAh g^−1^ at 0.1, 0.2, 0.5, and 1.0 C, respectively. When the current was set at 0.1 C, the specific capacity recovered to 767.8 mAh g^−1^.^[^ ^44^ ^]^ Schematic illustration of catalytic conversion from LPS to Li_2_S_2_/Li_2_S by the “softness unit”, NiNCs, in Al/M′‐M5‐3h‐S model is shown in Figure 4g. The radar map of the initial and final capacities of all electrode materials illustrated in Figure 4h shows the initial and final capacities of all electrode materials at 0.5 C, demonstrating the high electrochemical stability of Al/Ni‐M5‐3h‐S. To compare the improved cyclic performance of the Al/Ni‐M5‐3h‐S electrode, Table S5 of the Supporting Information displays the contrastive specific capacity in contrast with other MOF‐based materials as sulfur host. The “hardness unit” of amorphous Al_2_O_3_ was demonstrated through SEM images of the cycled cathodes after 200 cycles at 0.5 C, as shown in Figure S31 of the Supporting Information. The microstructure of Al/M′‐M5‐3 h maintained its initial morphology. In particular, the introduction of the second metal ions is beneficial for the preservation of the shape owing to the high residual stress.^[^ ^45^ ^]^ The comparison of the hardness of the amorphous Al_2_O_3_ in the framework nanocomposites with other MOF‐derived materials is listed in Table S6 of the Supporting Information.

To indirectly verify the catalytic activity of the “softness unit”, NiNCs, we soaked the powders of Al/Ni‐M5‐3 h in 4 mol L^−1^ HCl for 24 h to dissolve the NiNCs, and the obtained sample was named Al/Ni‐M5‐3h‐H (H denotes HCl). TEM images before and after pickling are shown in Figure S32a,b of the Supporting Information. It can be observed that after pickling, the NiNCs inside the Al/Ni‐M5‐3 h disappeared. Furthermore, the powder did not exhibit the magnetism of the NiNCs after pickling (Figure S32c,d, Supporting Information). The SEM images of Al/Ni‐M5‐3h‐H and the sulfur‐loaded sample, named Al/Ni‐M5‐3h‐H‐S, are shown in Figure S33a,b of the Supporting Information. The TEM image of Al/Ni‐M5‐3h‐H‐S illustrated in Figure S33c shows that sulfur was impregnated into porous Al/Ni‐M5‐3h‐H. The XRD patterns (Figure S33d, Supporting Information) show that the diffraction peaks of metallic Ni almost disappeared after pickling and only exhibited the peaks of amorphous carbon. Strong diffraction peaks of sulfur appeared after sulfur loading. The cyclic performance test (Figure S33e, Supporting Information) demonstrated that the specific capacity of Al/Ni‐M5‐3h‐H‐S decreased significantly.

**Figure 5:**
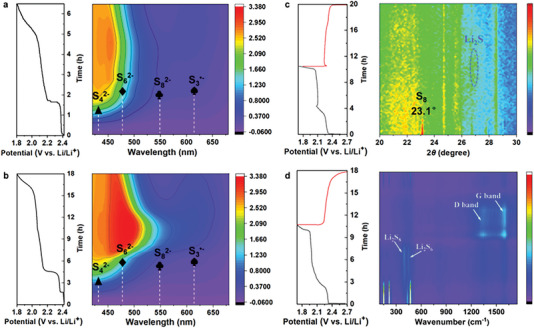
Electrochemical Contour maps of in situ UV/vis spectra and the discharge profiles of a) Al/Ni‐M5‐S electrode, b) Al/Ni‐M5‐3h‐S electrode. c) Contour map of in situ XRD patterns and the GCD profile of Al/Ni‐M5‐3h‐S electrode. d) Contour map of in situ Raman spectra the GCD profile of Al/Ni‐M5‐3h‐S.

To gain deeper insight into the discharge mechanism, in situ UV/vis technique was used to investigate the LPS conversion occurring in the electrolyte during the discharge (0.05 C) process. Various LPS were qualitatively and semiquantitatively determined through UV/vis spectroscopy (**Figure** **5**a,b). The UV/vis spectrum of the Al/Ni‐M5‐3h‐S electrode indicated a higher concentration of S_6_^2−^ compared to the Al/Ni‐M5‐S electrode owing to the fast redox reaction between Al/Ni‐M5‐3 h and LPS. Meanwhile, the in situ Li–S visual cuvette cell with the Al/Ni‐M5‐3h‐S electrode exhibited a high level of trisulfur radical (S_3_^•−^) concentration. This indicates an effective catalytic reaction from S_6_^2−^ to 2S_3_^•−^ and further verifies the discharge mechanism of S_8_ to Li_2_S_2_ or Li_2_S. The in situ XRD patterns of Al/Ni‐M5‐3h‐S during the discharge (0.1 C) process are shown in Figure 5c, where the peak at 23.1° is attributed to the S_8_ molecules. As the discharge depth increases, the peak intensity decreases. The peak at ≈27.1° was attributed to the final product of Li_2_S, which emerged at the end of the discharge process and disappeared as the charge process progressed. The in situ Raman technology was used to further demonstrate the behavior of catalysis during the operation of Li–S cells. The Raman spectra indicated the transition of S_8_ denoted by three major peaks at 143, 206, and 457 cm^−1^ to Li_2_S_8_ and Li_2_S_6_ denoted by the peaks at 396 and 443 cm^−1^ during the discharge process. In the case of the Al/Ni‐M5‐3h‐S cathode on the Cu mesh at 0.1 C (Figure 5d), strong S_8_ peaks were observed at the beginning of the discharge process. These peaks gradually disappeared as the discharge depth increased, indicating the complete conversion of S_8_ to LPS. By the end of the charging process, the peaks of S_8_ molecules reappeared, demonstrating the reversibility of S_8_→LPS→S_8_.

## 3 Conclusion

In this study, we prepared nanocomposites using diverse metal ion (Mn^2+^, Co^2+^, Ni^2+^, and Zn^2+^)‐introduced Al‐MIL‐96 as precursors. Owing to the pyrolysis‐generated Al_2_O_3_ from the parent Al‐MIL‐96, the obtained nanocomposites exhibited a hard framework and chemical stability. In particular, the Ni^2+^‐introduced Al‐MIL‐96‐derived nanocomposite alternated the “hardness unit”, amorphous Al_2_O_3_ framework, with the “softness unit”, NiNCs, as the sulfur host through a confined self‐reduction method. The existence of NiNCs was proved through XAFS, XPS, and XRD. The electrochemical performance test demonstrated that the NiNCs can significantly promote cyclic stability. In situ UV/vis analysis proved that the Al/Ni‐M5‐3h‐S electrode had an effective catalytic effect on LPS conversion. The electrode reaction mechanism during the GCD process was analyzed by in situ XRD and Raman spectroscopy. In addition, we prepared an Al/Ni‐M5‐3h‐H‐S electrode without NiNCs to demonstrate the catalytic reaction of NiNCs using an indirect method. Hence, these results indicated that the confined self‐reduction synthetic route is a rational approach for preparing host materials with catalytic effect. It is expected that this method could be extended to prepare sulfur host materials with catalytic effect on LPS conversion.

## Conflict of Interest

The authors declare no conflict of interest.

## Supporting information

Supporting Information

## Data Availability

Research data are not shared.
