# Supplementary material for: Confined Synthesis of Amorphous Al2O3 Framework Nanocomposites Based on the Oxygen‐Potential Diagram as Sulfur Hosts for Catalytic Conversion
Source: Adv Sci (Weinh). 2023 Jun 19;10(24):2302215. doi: 10.1002/advs.202302215 (PMC10460837; doi:10.1002/advs.202302215)
Supplement: Supplementary file 1 — Supporting Information [file ADVS-10-2302215-s001.pdf]

ADVANCED SCIENCE
Open Access

Supporting Information

for *Adv. Sci.*, DOI 10.1002/advs.202302215

Confined Synthesis of Amorphous Al_2O_3 Framework Nanocomposites Based on the Oxygen-Potential Diagram as Sulfur Hosts for Catalytic Conversion

*Pengbiao Geng, Yuxing Lin, Meng Du, Chunsheng Wu, Tianxing Luo, Yi Peng, Lei Wang, Xinyuan Jiang, Shuli Wang, Xiuyun Zhang, Lubin Ni, Shuangqiang Chen, Mohsen Shakouri and Huan Pang**

Supplementary information

Confined Synthesis of Amorphous Al_2O_3 Framework Nanocomposites Based on the Oxygen-potential Diagram as Sulfur Hosts for Catalytic Conversion

*Pengbiao Geng, Yuxing Lin, Meng Du, Chunsheng Wu, Tianxing Luo, Yi Peng, Lei Wang, Xinyuan Jiang, Xiuyun Zhang, Lubin Ni, Shuangqiang Chen, Mohsen Shakouri, and Huan Pang**

P. Geng, M. Du, C. Wu, T. Luo, Y. Peng, X. Jiang, Prof. L. Ni, Prof. H. Pang
School of Chemistry and Chemical Engineering, Yangzhou University, Yangzhou, Jiangsu, 225009, P. R. China
E-mail: huanpangchem@hotmail.com; panghuan@yzu.edu.cn

Y. Lin, Prof. X. Zhang
College of Physics Science and Technology, Yangzhou University, Yangzhou, Jiangsu, 225009, P. R. China

L. Wang, Prof. S. Chen
Department of Chemical Engineering, School of Environmental and Chemical Engineering, Shanghai University, Shanghai 200444, P. R. China

Dr. M. Shakouri
Canadian Light Source Inc., University of Saskatchewan, Saskatoon, S7N 2V3, Canada

Keywords: confined synthesis, oxygen-potential diagram, amorphous aluminum oxide, Ni nanocrystals, catalysis

Synthesis of Al-MIL-96

Al-MIL-96 were prepared using co-solvent method. Typically, 5 mmol 1,3,5-benzenetricarboxylic acid (BTC) and 5 mmol aluminum nitrate nonahydrate were successively dissolved in 60 mL mixture solution of 30 mL N,N-dimethylformamide (DMF)/30 mL deionized water. The resulting solution was transferred to a 100 mL Teflon high-pressure autoclave. After sealing, the autoclave was kept at 160 °C for 24 h, and then cool down to room temperature. The precipitate was collected by centrifugation, and washed several times with deionized water and methanol. Then the product was heated at 50 °C under vacuum in a drying oven for 24 h to remove the solvent in the MOF.

Synthesis of Al/M'-M*x* (*x* = 1, 5, and 10) and Al/M'-M5-S

The bimetallic Al/M'-M*x* samples were synthesized by the same method as for Al-MIL-96 except that a certain amount of nitrates of Mn^{2+}, Co^{2+}, Ni^{2+}, and Zn^{2+} was added together with aluminum nitrate nonahydrate into the solution. The molar content of BTC is the same as Al^{3+} and second metal. Sublimed sulfur was introduced into MOFs through a melt-diffusion method at 155 °C. Taking Al-MIL-96 with 60 ω% sublimed sulfur-loading (Al-MIL-96-S) as an example, 100 mg of Al-MIL-96 powder was firstly grinded together with 150 mg of sublimed sulfur into a fine mixture that was transferred into a 25-mL Teflon-line sealed autoclave, and heated at 155 °C for 12 h to generate the Al-MIL-96-S sample. The other MOF crystals with 60 ω% sublimed sulfur-loading samples were obtained similarly.

Synthesis of Al/M'-M5-3h and the sulfur-loaded sample

The dried Al/M'-M5 were heated to 700 °C with a heating rate of 3 °C min^{-1} in an N_2 atmosphere and keep 700 °C for 3 h, then directly cooled down to room temperature. The sulfur-loading process is the same as the Al/M'-M5-S except the sulfur content of 70%.

Synthesis of Al/Ni-M5-3h-H and Al/Ni-M5-3h-H-S

To remove the NiNCs in Al/Ni-M5-3h, 4 mol/L HCl were prepared, and then 60 mg Al/Ni-M5-3h was added for soaking 24 h. The precipitate was collected by centrifugation, and washed several times with deionized water and alcohol. Then the product was heated at 50 °C under vacuum in a drying oven for 24 h. The sulfur-loading process and sulfur content are the same as the Al/M'-M5-3h-S.

Characterization

The XRD patterns were performed by Rigaku MiniFlex 600 with Cu Kα radiation of 40 KV (λ = 1.5418 Å). SEM images were obtained by Zeiss-Supra 55 microscope at an acceleration voltage of 5 KV. TEM and EDX elemental mapping scans were recorded using Tecnai G2 F30 S-TWIN at an acceleration voltage of 300 KV. The N_2 adsorption-desorption isothermals were obtained by Autosorb-IQ3. Electron paramagnetic resonance (EPR) spectra were recorded on a Bruker A300 electron paramagnetic resonance spectrometer at room temperature. Raman spectra were obtained via INVIA REFLEX (Renishaw), in the range 150−4000 cm^{-1}. XPS analysis was carried out using Thermo Scientific ESCALAB 250Xi X-ray photoelectron spectrometer with Al Kα radiation as the excitation source. The survey thickness is 2−3 nm. The accurate sulfur mass on each electrode was calculated according to TGA curves under N_2 flow, 5 °C /min, and the elemental analysis data from Elementar, VarioELcube Co. (C, H, N, S mode). XAFS measurements of Al/M'-M5 were performed in Canadian Light Source, Saskatoon, Canada. XAFS measurements of Al/Ni-M5-3h and Al/Ni-M5-3h-S were performed in Shanghai Synchrotron Radiation Facility.

Adsorption and soaking tests

For the adsorption test, 20 mg MOFs were soaked in 3 mL Li_2S_4 solution (10 mmol/L). The Li_2S_4 solution were prepared according to the reaction Equation S1:

$$3S + Li_2S \leftrightarrow Li_2S_4 \qquad (S1)$$

using a solvent mixture of 1,3-dioxolane (DOL) and dimethoxyethane (DME) (1:1 in volume). UV/Vis spectra of the above solutions (diluted 5 times before testing) were recorded by using a UV2550 instrument (Shimadzu, Japan). The concentration variations in these solutions was detected by the UV/Vis spectrocopy.

DFT Computational methods and structures

The MIL-96-Al (002) surface was represented as a theoretical calculation model. All structure relaxations calculations within spin-polarized DFT were executed in the Vienna *ab initio* simulation package (VASP)[1]. The electron exchange−correlation interactions were described by the Perdew−Burke−Ernzerhof (PBE) functional within the generalized gradient approximation (GGA)[2]. A cutoff energy of 350 eV was adopted for the plane-wave basis and the k-points were sampled using 1 × 1 × 1 Monkhorst–Pack mesh. To eliminate the possible interactions between neighboring slab layers, a vacuum layer with a thickness of 15.0 Å was set along the nonperiodic *c*-axis direction. The energy and force convergence thresholds for the iteration in the self-consistent field (SCF) were set to 0.01 eV and 0.01 eV $Å^{-1}$, respectively. The adsorption energy of Li_2S_4 and S_8 (E_{ads}) was calculated by:

$$E_{ads} = E_{[\mathrm{substrate+\ Li_2S_4/S_8}]} - E_{\mathrm{substrate}} - E_{\mathrm{Li_2S_4/S_8}}$$

where $E_{[\mathrm{substrate+Li_2S_4/S_8}]}$, $E_{\mathrm{substrate}}$, and $E_{\mathrm{Li_2S_4/S_8}}$ are the total energies of the adsorbed–substrate complex, the substrate, and the isolated Li_2S_4 or S_8, respectively.

Li−S cell assembling and test

Electrode preparation: The slurry was mixed with sulfur-loaded sample, Super P, and polyvinylidene fluoride (PVDF) in a weight ratio of 7:2:1 in N-methyl-2-pyrrolidone as dispersant. The slurry was cast on the Al foil, and dried overnight at 50 °C under vacuum. The obtained working electrodes were cut to circular electrode with a diameter of 12 mm. The mass loading of active sulfur was *ca.* 1.1 mg cm^{-2}. The CR2032-type coin cells were fabricated using the working electrode, lithium foil as the counter and anode electrode,

Celgard 2400 as the separator. The electrolyte was used 1.0 M lithium bis(trifluoromethanesulfonyl)imide (LiTFSI Sigma-Aldrich (USA), 99.95%) in 1,3-dioxolane (DOL, Sigma-Aldrich (USA), 99.0%) and 1,2-dimethoxyethane (DME, Sigma-Aldrich (USA), 99.0% (volume ratio, 1:1) with 1 ω% $LiNO_3$ in an argon-filled glove box (where both water and oxygen levels are below 0.1 ppm. The value of the electrolyte to S (E/S) ratio is *ca.* 18 μL mg^{-1} (according to the weight of S). The GCD tests were estimated in the voltage window of 1.7−2.7 V. The rate capability was also tested by varying the current density from 0.1 C to 1 C (1 C = 1675 mA g^{-1}) on a battery measurement system (CT3001A, Wuhan Land, China) at room temperature. CV and EIS curves were measured on an electrochemical workstation (CHI660E, Chenhua, Shanghai, China).

***In situ* UV/Vis measurement**

The cathode slurry comprised of an active material powder Al/Ni-M5-S or Al/Ni-M5-3h-S, Super P, and PVDF with a mass ratio of 7:2:1. Then, the nickel foam was selected as the collector (1 × 0.6 cm^2). The sulfur mass loading on the electrode was *ca.* 6 mg cm^{-2}, and the current density is 0.05 C. The *in situ* UV/Vis cells were assembled using Al/Ni-M5-S or Al/Ni-M5-3h-S electrode as the cathodes and Li metal as the anode, using a custom made *in situ* cuvette. The *in situ* cuvette cell was assembled in an Ar filled glove box and sealed in 3 mL of Li−S electrolyte. UV/Vis absorption spectra (UV/Vis, Shimadzu UVmini-1280 spectrophotometer) were used to detect the concentration and elemental chemical states of the LPS.

***In situ* XRD measurement**

In situ XRD was performed on X-ray diffractometer (MiniFlex600-C) and a cell mould with Be. The mass ratio of slurry is the same as *in situ* UV/Vis test, and sulfur area mass loading on the electrode was 1.3 mg cm^{-2}, and the current density is 0.1 C. The current collector is Al foil.

***In situ* Raman measurement**

In situ Raman was performed on Raman spectroscopy (NVIA) and a cell mould with glass, The mass ratio of slurry is the same as *in situ* UV/Vis test, and the sulfur area mass loading on the electrode was 1.2 mg cm^{-2}, and the current density is 0.1 C. The current collector is Cu mesh.

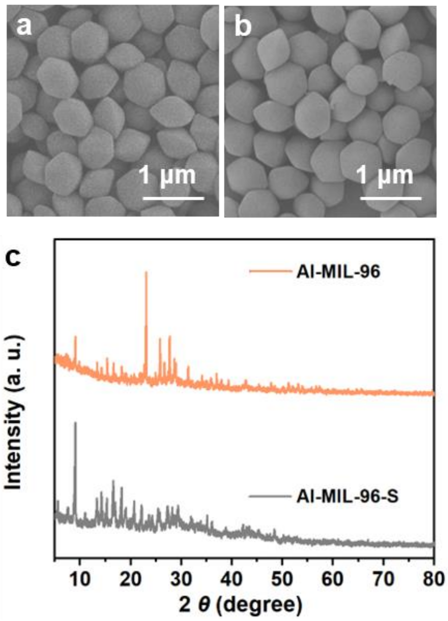

Figure S1. SEM images of (a) Al-MIL-96, and (b) Al-MIL-96-S; (c) XRD patterns of Al-MIL-96 and Al-MIL-96-S. After sulfur loading, the hexagonal bipyramidal shape can be keeped.

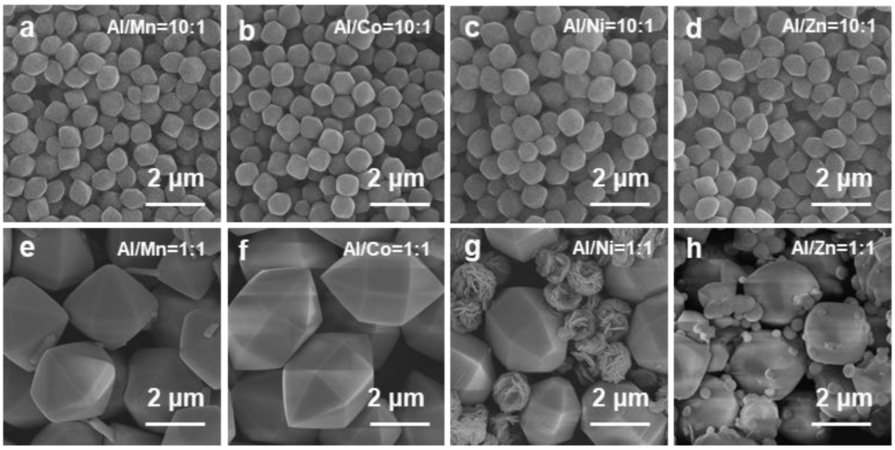

Figure S2. SEM images of (a−d) Al/M'-M1, and (e−h) Al/M'-M10.

Table S1. ICP-OES Analyses of Al/M'-M*x*.

Samples	Al:M'	Molar ratio (Used)	Molar ratio (Detected)
**Al/Mn-M1**	Al:Mn	10:1	12.60:0.18
**Al/Mn-M5**	Al:Mn	5:1	11.90:1.72
**Al/Mn-M10**	Al:Mn	1:1	11.49:3.52
**Al/Co-M1**	Al:Co	10:1	10.71:0.82
**Al/Co-M5**	Al:Co	5:1	10.93:2.02
**Al/Co-M10**	Al:Co	1:1	12.09:6.98
**Al/Ni-M1**	Al:Ni	10:1	12.31:1.83
**Al/Ni-M5**	Al:Ni	5:1	11.46:2.86
**Al/Ni-M10**	Al:Ni	1:1	11.73:7.23
**Al/Zn-M1**	Al:Zn	10:1	12.23:0.17
**Al/Zn-M5**	Al:Zn	5:1	12.86:4.71
**Al/Zn-M10**	Al:Zn	1:1	11.99:9.03

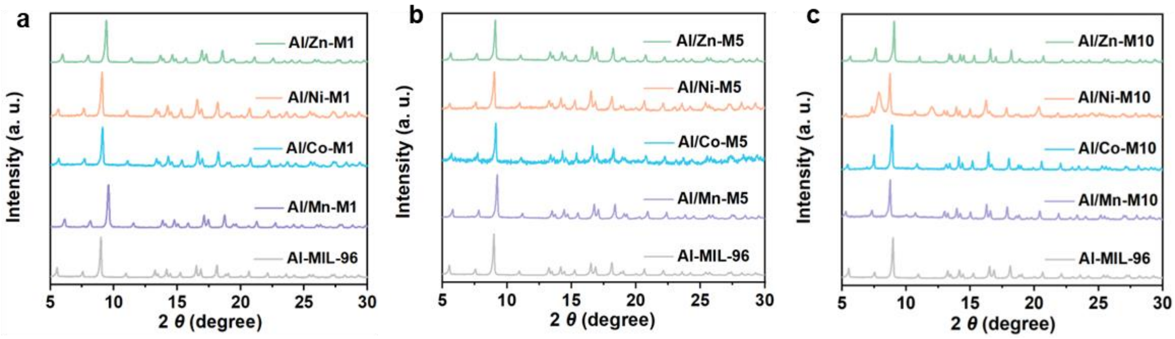

Figure S3. XRD patterns of (a) Al/M'-M1, (b) Al/M'-M5, and (c) Al/M'-M10.

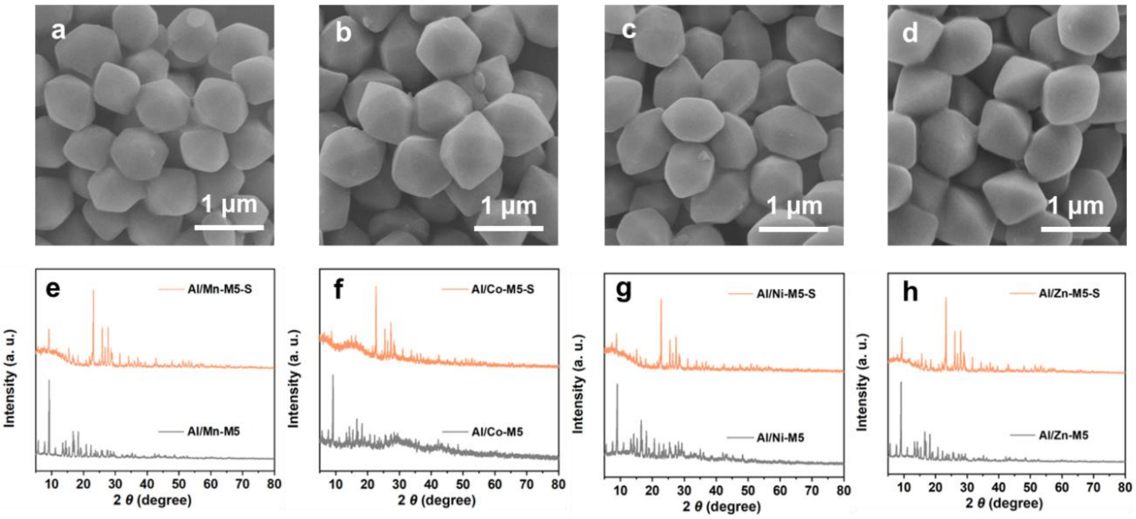

Figure S4. SEM images of (a) Al/Mn-M5-S, (b) Al/Co-M5-S, (c) Al/Ni-M5-S, and (d) Al/Zn-M5-S; XRD patterns of (e) Al/Mn-M5 and -S, (f) Al/Co-M5 and -S, (g) Al/Ni-M5 and -S, (h) Al/Zn-M5 and -S.

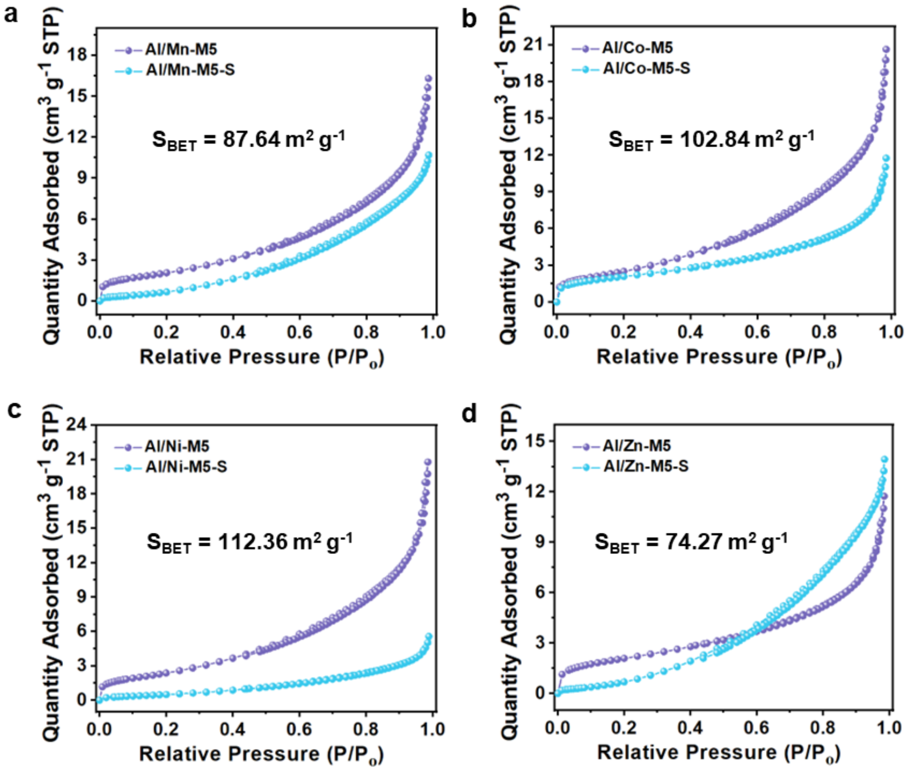

Figure S5. N_2 adsorption-desorption analysis of (a) Al/Mn-M5 and -S, (b) Al/Co-M5 and -S, (c) Al/Ni-M5 and -S, (d) Al/Zn-M5 and -S, the S_{BET} values belong to Al/M'-M5.

Table S2. Pore volume results given by N_2 adsorption analysis, and corresponding theoretical sulfur-loaded amount. Sulfur density = 2.36 g/cm^3.

**Samples**	**Pore volume/ cc g^{-1}**	**Theoretical sulfur-loaded amount/ g g^{-1}**
**Al-MIL-96**	0.75	1.77
**Al/Mn-M5**	0.56	1.32
**Al/Co-M5**	0.62	1.46
**Al/Ni-M5**	0.67	1.58
**Al/Zn-M5**	0.59	1.39

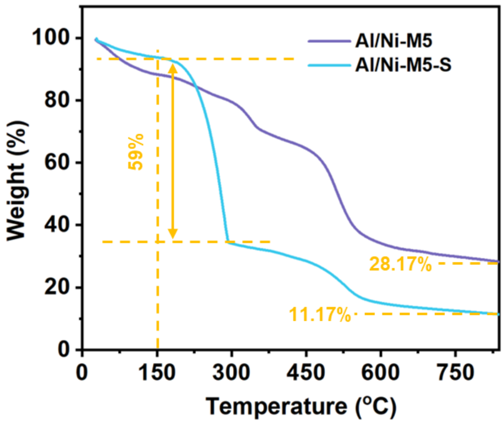

Figure S6. TGA curves of Al/Ni-M5 and -S.

Table S3. Elemental analysis data (mass fraction) of the sulfur-loaded samples.

**Samples**	**N %**	**C %**	**S %**
**Al-MIL-96-S**	1.124	13.430	59.189
**Al/Mn-M5-S**	1.325	13.922	59.092
**Al/Co-M5-S**	1.439	14.035	60.095
**Al/Ni-M5-S**	1.345	13.931	60.101
**Al/Zn-M5-S**	1.397	14.102	59.925

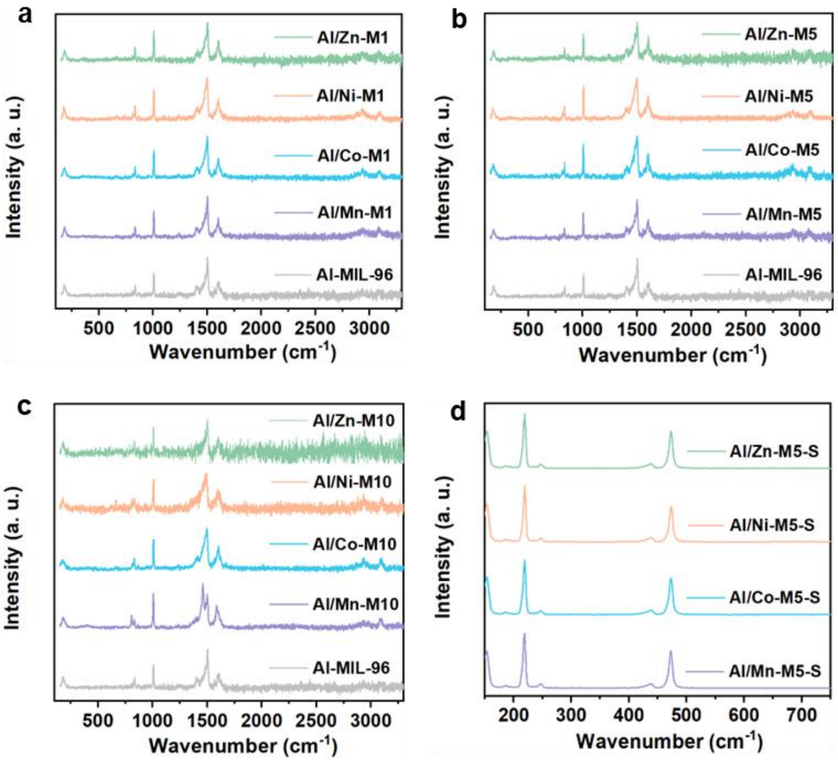

Figure S7. Raman spectra of (a) Al/M'-M1, (b) Al/M'-M5, (c) Al/M'-M10, and (d) Al/M'-M5-S.

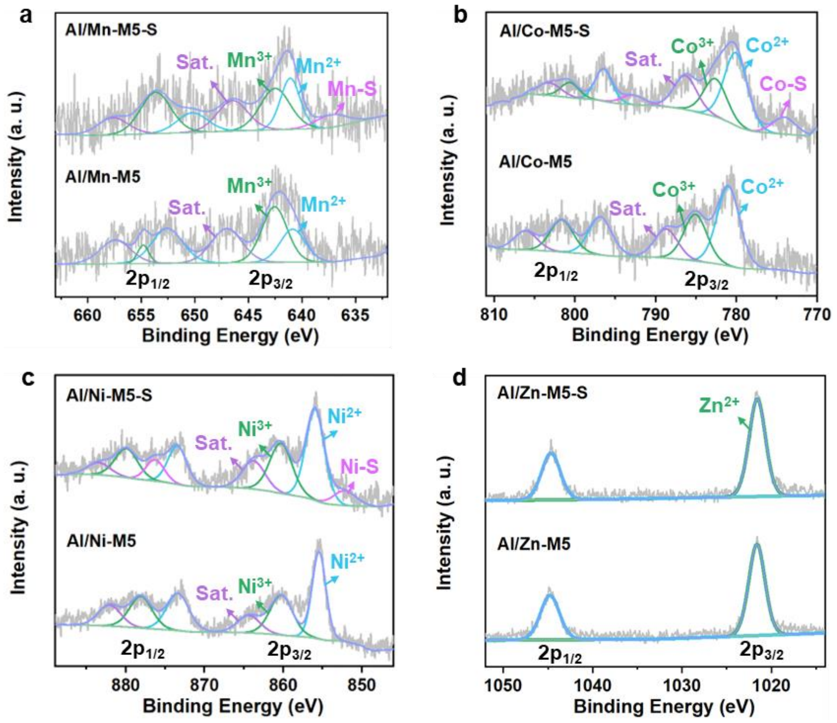

Figure S8. (a) Mn 2p XPS spectra of Al/Mn-M5 and -S; (b) Co 2p XPS spectra of Al/Co-M5 and -S; (c) Ni 2p XPS spectra of Al/Ni-M5 and -S; (d) Zn 2p XPS spectra of Al/Zn-M5 and -S.

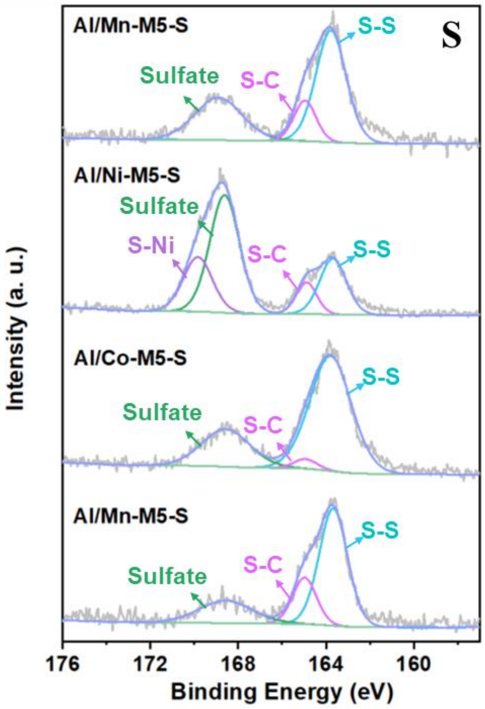

Figure S9. S 2p XPS spectra of Al/M'-M5-S.

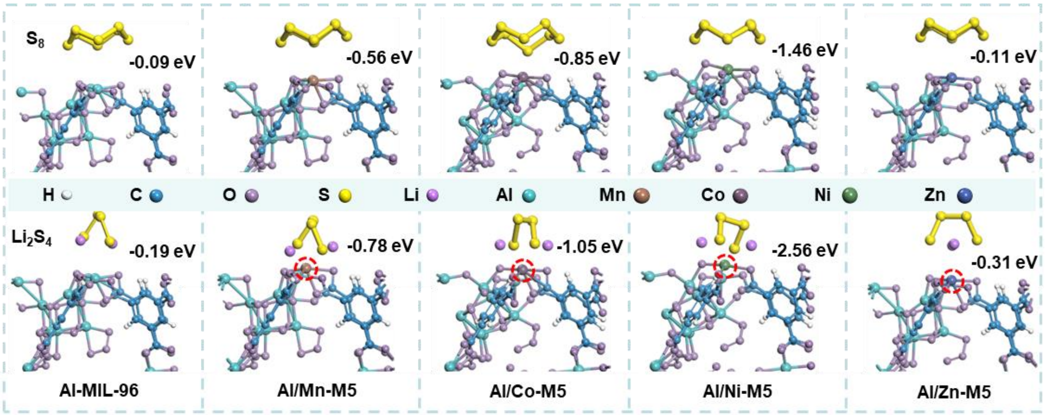

Figure S10. DFT calculations of the optimized geometric structures of adsorping surfaces.

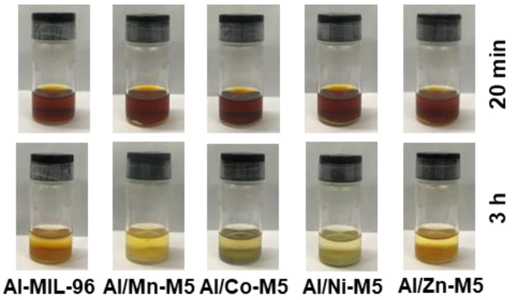

Figure S11. Optical photographs of Li_2S_4 solution permeation tests of Al-MIL-96 and Al/M'-M5 crystals at 20 min and 3 h, respectively.

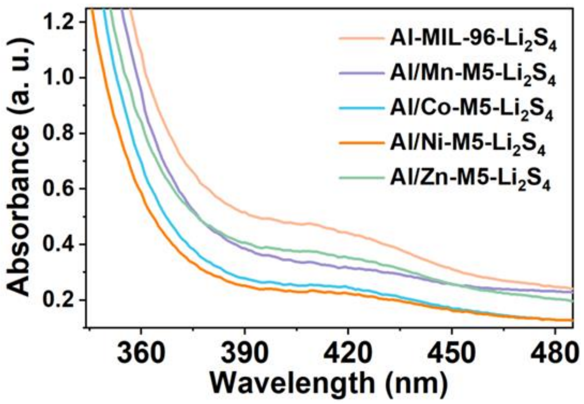

Figure S12. Comparison of the UV/Vis absorption spectra of Al-MIL-96 and Al/M'-M5 supernatants at 3 h.

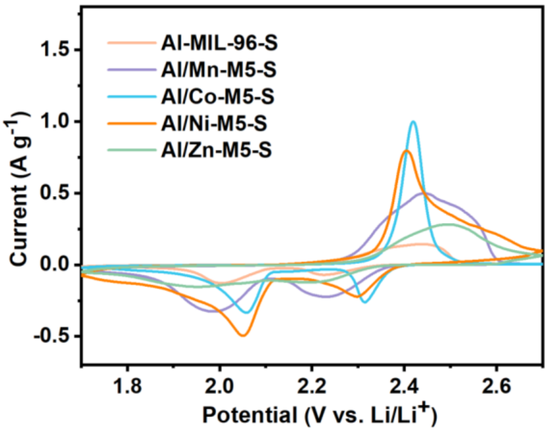

Figure S13. Comparison of the CV curves during third cycle of Al/M'-M5-S at 0.1 mV s^{-1}.

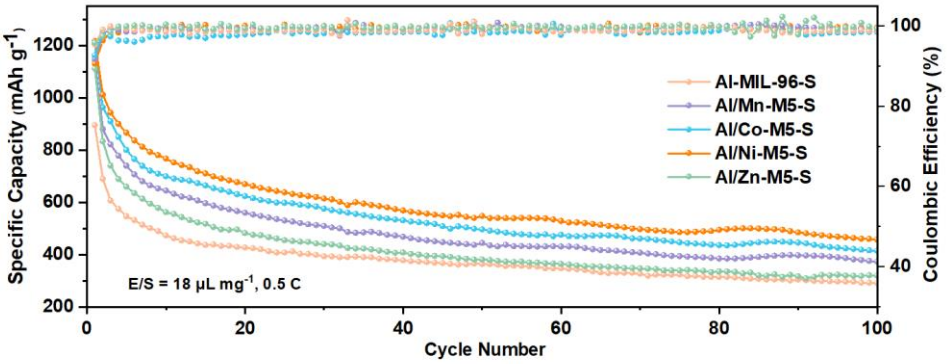

Figure S14. Cyclic performance of Al-MIL-96-S and Al/M'-M5-S at 0.5 C.

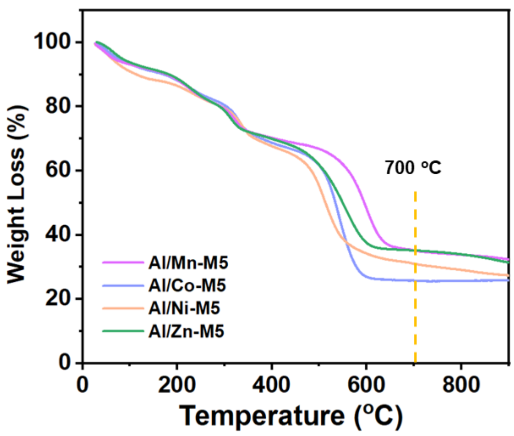

Figure S15. TGA curves of Al/M'-M5.

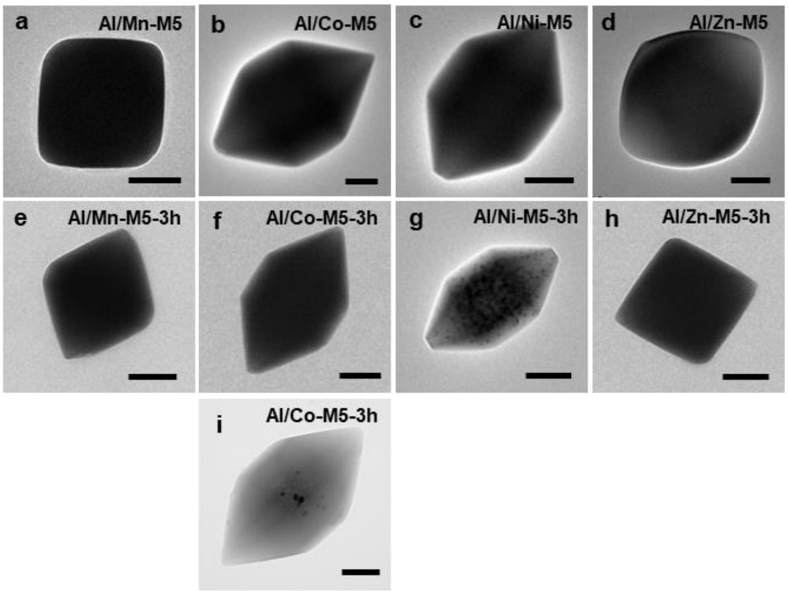

Figure S16. TEM images of (a−d) Al/M'-M5; (e−h) Al/M'-M5-S, Figure i is contrast-adjusted TEM image of Al/Co-M5-3h.

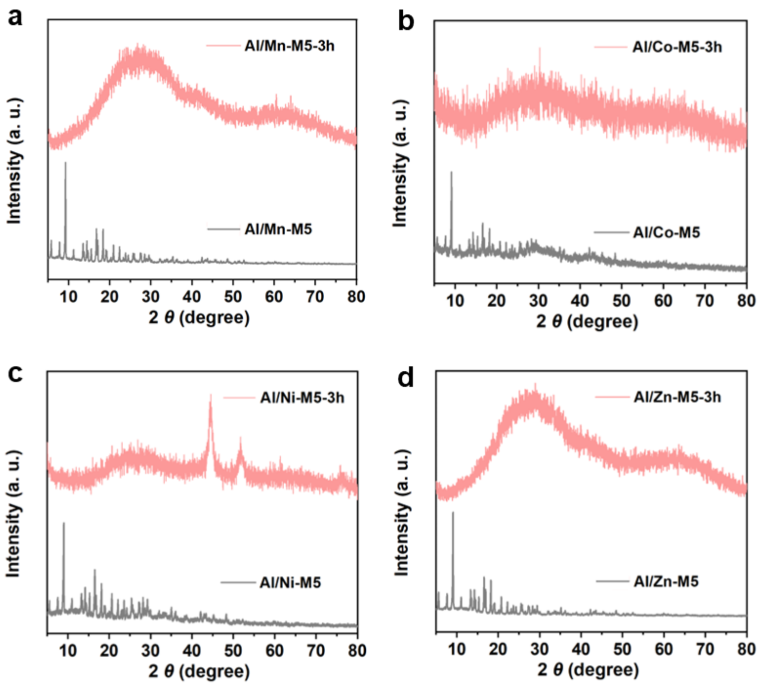

Figure S17. XRD patterns of Al/M'-M5 and -3h. The Al/Mn-M5-3h and Al/Zn-M5-3h have no distinct diffraction peaks, indicating their amorphous feature.

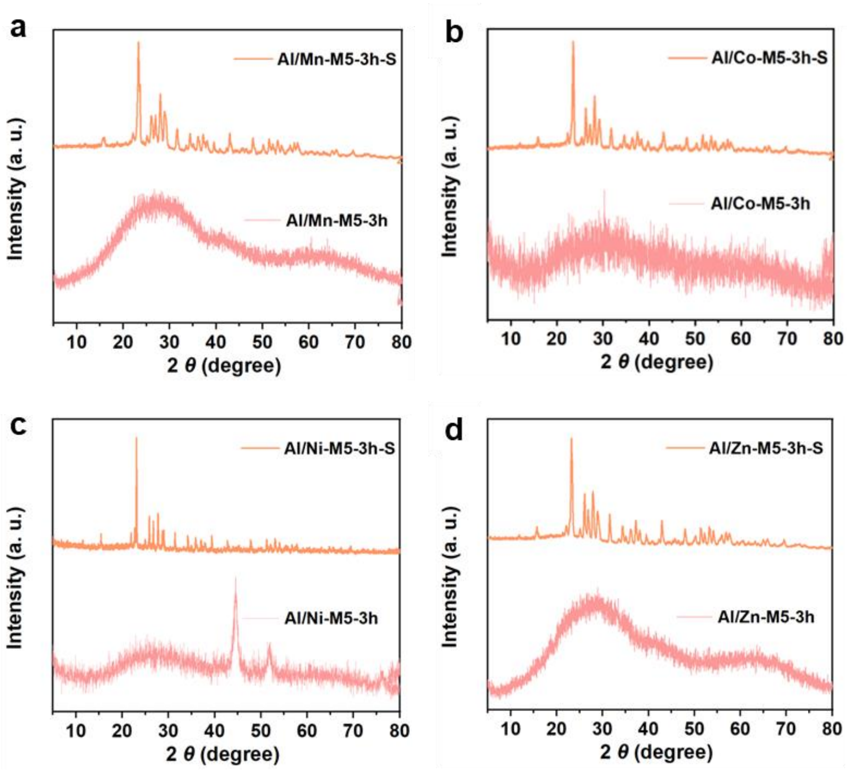

Figure S18. XRD patterns of Al/M'-M5-3h and -S.

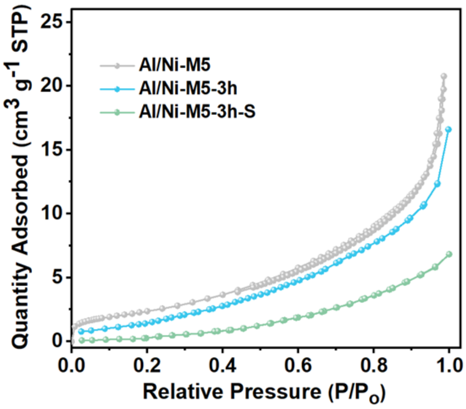

Figure S19. N_2 adsorption-desorption analysis of Al/Ni-M5, Al/Ni-M5-3h, and Al/Ni-M5-3h-S.

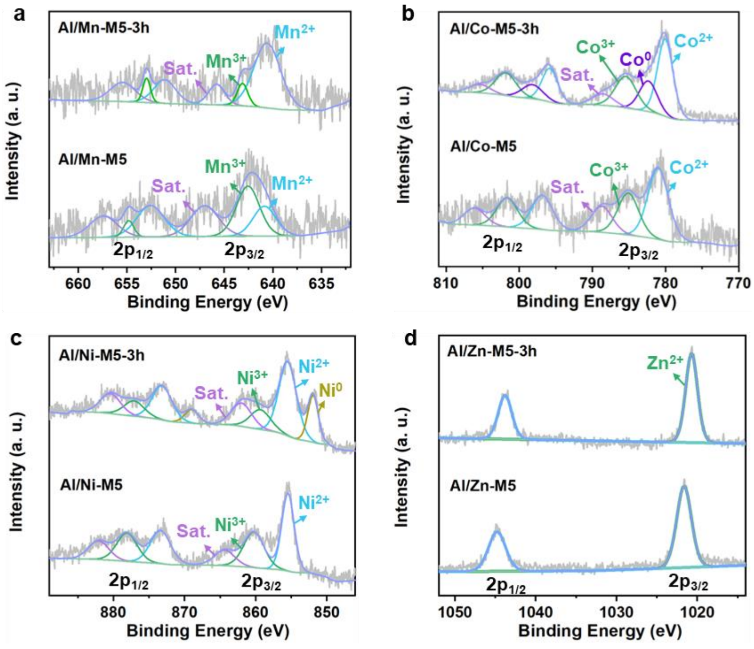

Figure S20. Mn, Co, Ni, and Zn 2p XPS spectra of Al/M'-M5 and -3h.

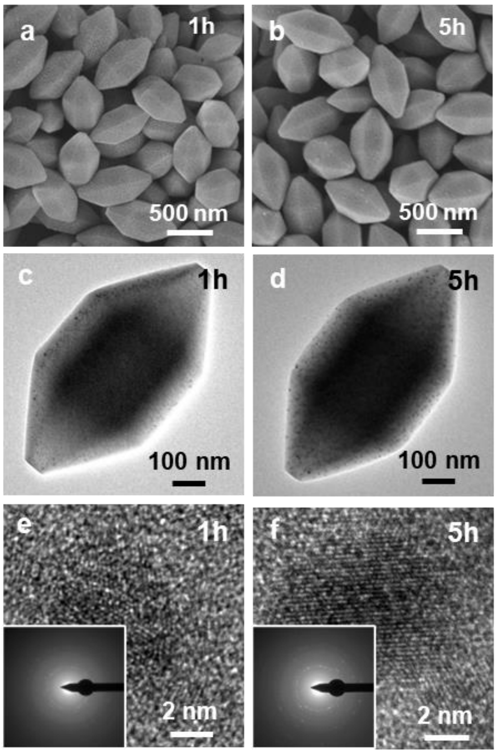

Figure S21. SEM images of (a) Al/Ni-M5-1h, (b) Al/Ni-M5-5h; TEM images of (c) Al/Ni-M5-1h, (d) Al/Ni-M5-5h; HRTEM images and SAED patterns of (e) Al/Ni-M5-1h, (f) Al/Ni-M5-5h.

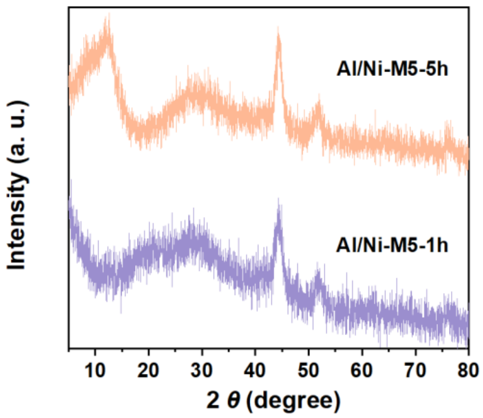

Figure S22. XRD patterns of Al/Ni-M5-1h and -5h.

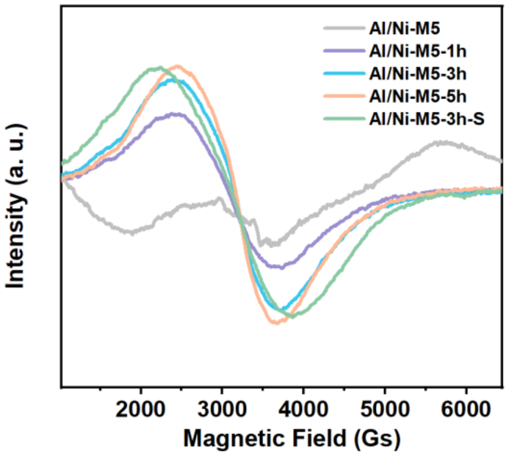

Figure S23. EPR spectrum of Al/Ni-M5, Al/Ni-M5-1h, Al/Ni-M5-3h, Al/Ni-M5-5h, and Al/Ni-M5-3h-S.

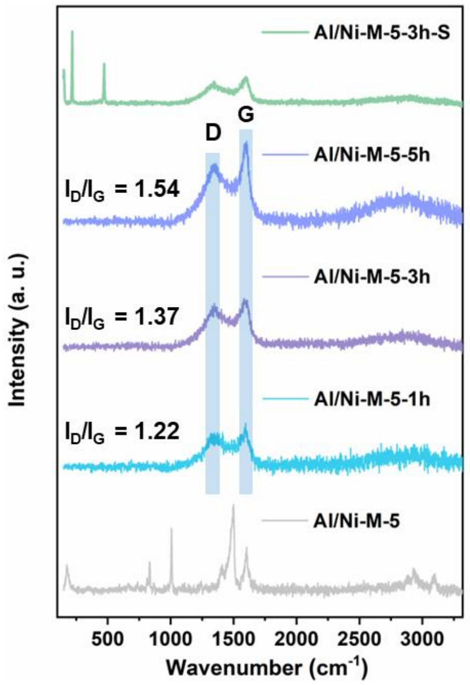

Figure S24. Raman spectra of Al/Ni-M5, Al/Ni-M5-1h, Al/Ni-M5-3h, Al/Ni-M5-5h, and Al/Ni-M5-3h-S.

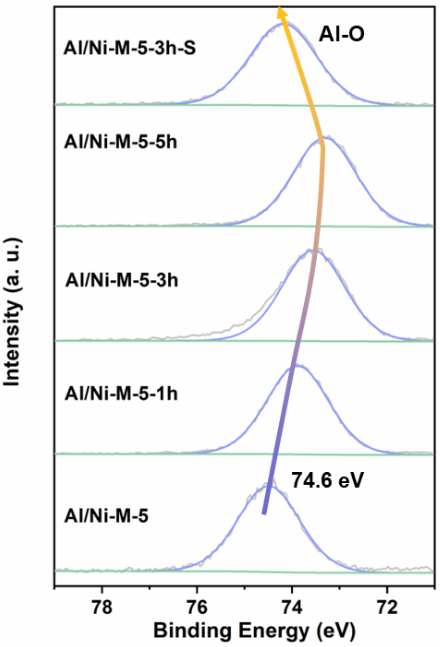

Figure S25. Al 2p XPS spectra of Al/Ni-M5, Al/Ni-M5-1h, Al/Ni-M5-3h, Al/Ni-M5-5h, and Al/Ni-M5-3h-S.

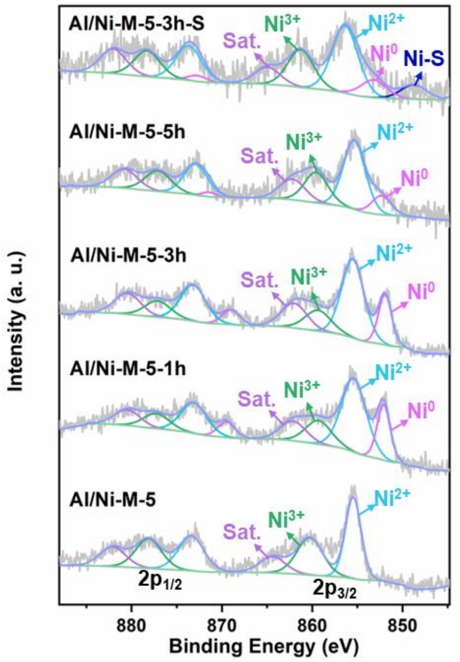

Figure S26. Ni 2p XPS spectra of Al/Ni-M5, Al/Ni-M5-1h, Al/Ni-M5-3h, Al/Ni-M5-5h, and Al/Ni-M5-3h-S.

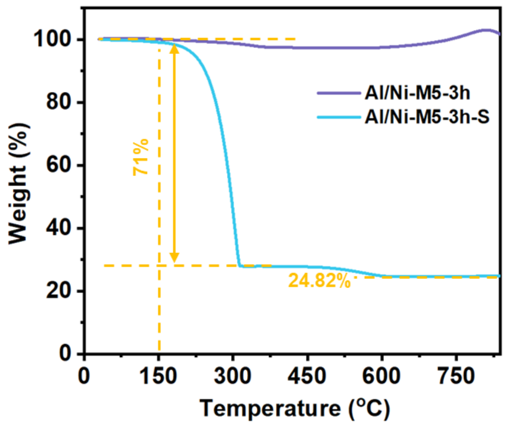

Figure S27. TGA curves of Al/Ni-M5-3h and -S.

Table S4. Elemental analysis data (mass fraction) of the sulfur-loaded samples.

Samples	N %	C %	S %
**Al/Mn-M5-3h-S**	0.060	8.749	70.870
**Al/Co-M5-3h-S**	0.033	6.976	69.925
**Al/Ni-M5-3h-S**	0.046	7.490	71.170
**Al/Zn-M5-3h-S**	0.061	8.899	70.925

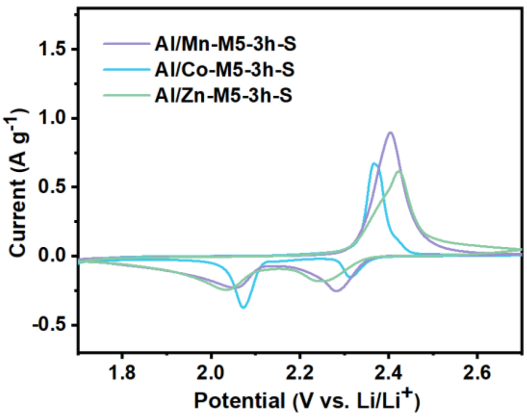

Figure S28. Comparison of the CV curves during third cycle of Al/Mn-M5-3h-S, Al/Co-M5-3h-S, and Al/Zn-M5-3h-S.

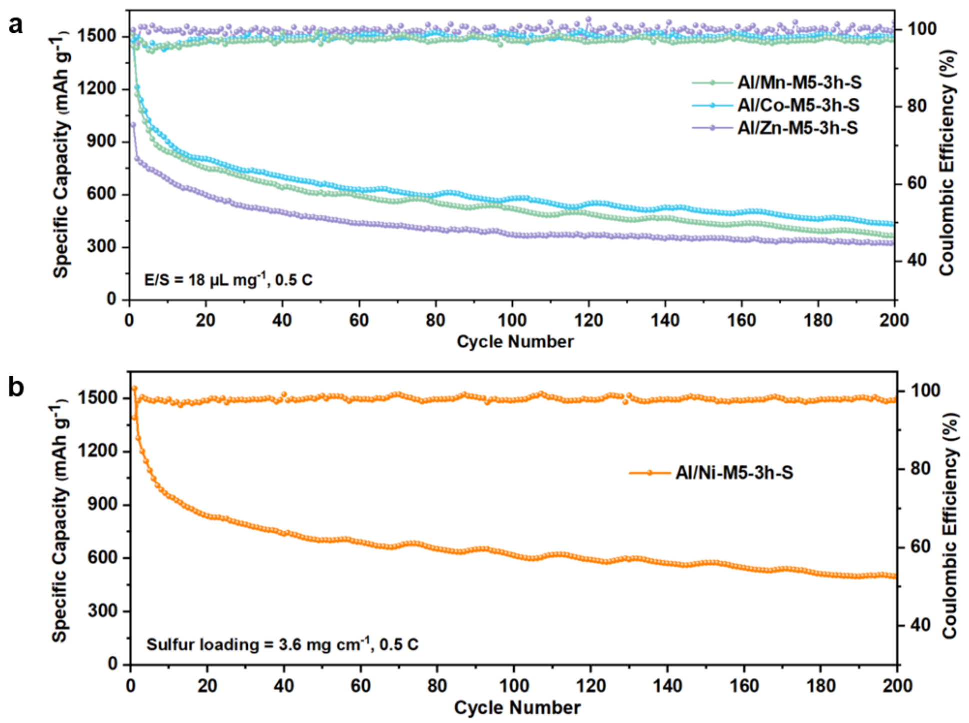

Figure S29. Cyclic performance of (a) Al/Mn-M5-3h-S, Al/Co-M5-3h-S, Al/Zn-M5-3h-S and (b) high sulfur loading mass of Al/Ni-M5-3h-S.

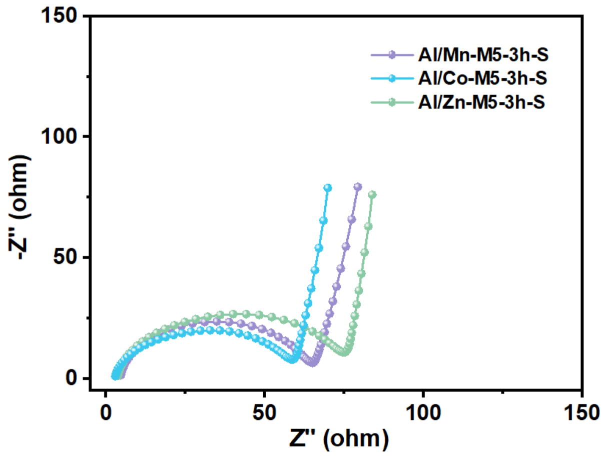

Figure S30. EIS result of Al/Mn-M5-3h-S, Al/Co-M5-3h-S, and Al/Zn-M5-3h-S.

Table S5. Comparison of the specific capacity for reported sulfur host.

**Host Materials**	**Initial Capacity (mAh g^{-1})**	**Cycles Number**	**Final Capacity (mAh g^{-1})**	**Discharge Rate (C)**	**Ref.**
S@HKUST-1	657	50	~573	0.2	[3]
Ni-MOF/S	689	100	611	0.1	[4]
S/NH_2-MIL-53	1125	70	436	0.1	[5]
MIL-100(Cr)/S	1580	60	~450	0.1	[6]
S/MOF-808	~590	300	~225	1.0	[7]
S-in-MIL-101	~795	80	~310	0.3	[8]
Al-MIL-96-S	907.5	200	227.7	0.5	[9]
**Al/Ni-M5-S**	**1216.8**	**200**	**356.6**	**0.5**	**This**
**Al/Ni-M5-3h-S**	**1556.8**	**200**	**496.0**	**0.5**	**This**

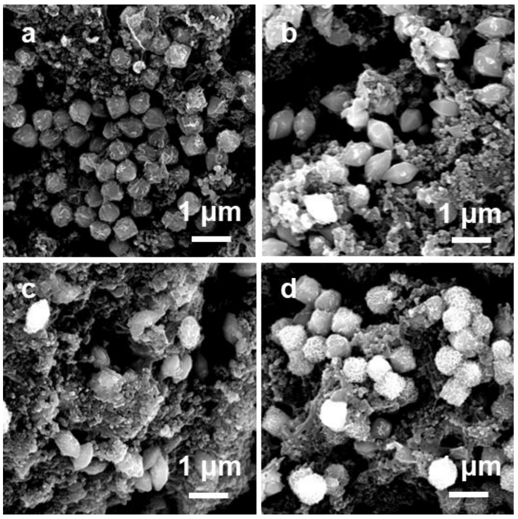

Figure S31. SEM images of (a) Al/Mn-M5-3h-S, (b) Al/Co-M5-3h-S, (c) Al/Ni-M5-3h-S, and (d) Al/Zn-M5-3h-S on Al foil after 200 GCD cycles at 0.5 C.

Table S6. Reports of MOF-derived morphologies.

Initial morphologies of MOFs	Sample names	Gas atmosphere, Temperature	MOF-derived morphologies	Ref.
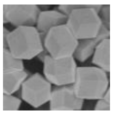	ZIF-67-Co	Ar/H_2, 700	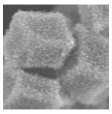	[10]
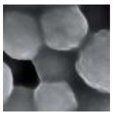	Ni-MOF	Ar, 450	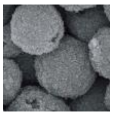	[11]
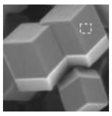	Zn-Co-ZIF	N_2, 400	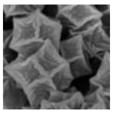	[12]
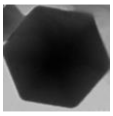	Fe-ZIF-8	Ar, 900	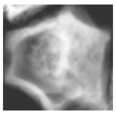	[13]
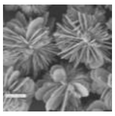	Ni-MOPh	Air, 600	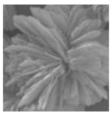	[14]
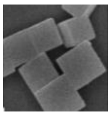	$Mn_3[Co(CN)_6]_2 \cdot 9H_2O$	Air, 475	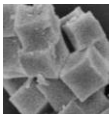	[15]
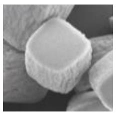	ZIF-9-Co	N_2, 800	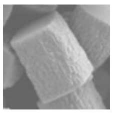	[16]
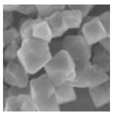	NENU-5	N_2, 800	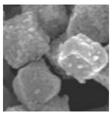	[17]
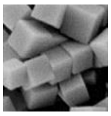	$Mn_3[Fe(CN)_6]_2 \cdot nH_2O$	Air, 600	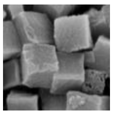	[18]
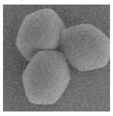	MIL-96-Al	N_2, 700	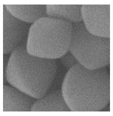	**Al-MIL-96**
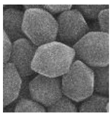	Al/Ni-M5	N_2, 700	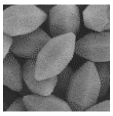	**Al/Ni-M5**

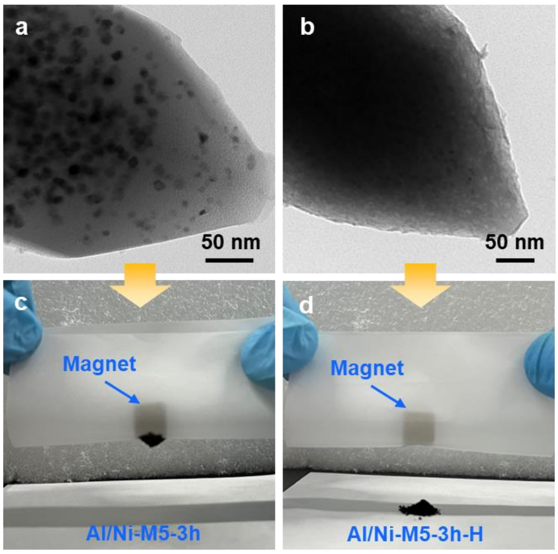

Figure S32. TEM images of (a) Al/Ni-M5-3h, (b) Al/Ni-M5-3h-H; Optical photos of (c) Al/Ni-M5-3h powder with magnet, (d) Al/Ni-M5-3h-H powder with magnet.

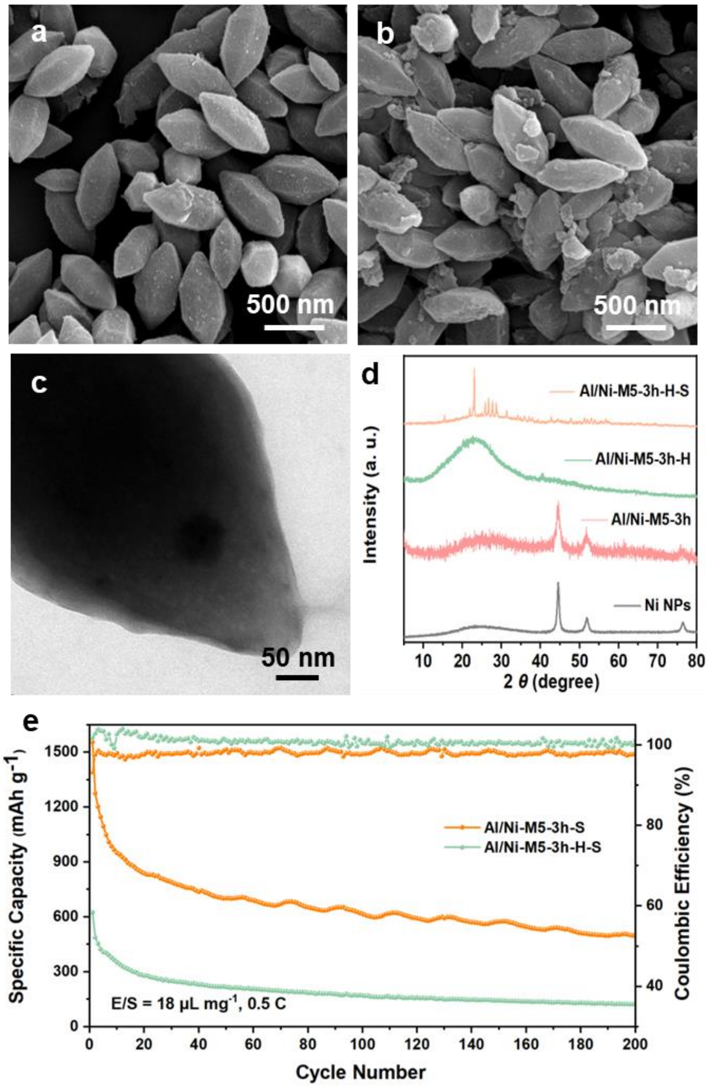

Figure S33. SEM images of (a) Al/Ni-M5-3h-H, (b) Al/Ni-M5-3h-H-S; (c) TEM image of Al/Ni-M5-3h-H-S; (d) XRD patterns of Al/Ni-M5-3h, Al/Ni-M5-3h-H, and Al/Ni-M5-3h-H-S; (e) Cyclic performance of Al/Ni-M5-3h-S, and Al/Ni-M5-3h-H-S.

References

[1] G. Sun, J. Kürti, P. Rajczy, M. Kertesz, J. Hafner, G. Kresse, *J. Mol. Struct. - Theochem* **2003**, *624*, 37.

[2] J. P. Perdew, K. Burke, M. Ernzerhof, *Phys. Rev. Lett.* **1996**, *77*, 3865.

[3] Y. Mao, G. Li, Y. Guo, Z. Li, C. Liang, X. Peng, Z. Lin, *Nat. Commun.* **2017**, *8*, 14628.

[4] J. Zheng, J. Tian, D. Wu, M. Gu, W. Xu, C. Wang, F. Gao, M. H. Engelhard, J.-G. Zhang, J. Liu, J. Xiao, *Nano Lett.* **2014**, *14*, 2345.

[5] J. Zhou, R. Li, X. Fan, Y. Chen, R. Han, W. Li, J. Zheng, B. Wang, X. Li, *Energy Environ. Sci.* **2014**, *7*, 2715.

[6] R. Demir-Cakan, M. Morcrette, F. Nouar, C. Davoisne, T. Devic, D. Gonbeau, R. Dominko, C. Serre, G. Férey, J.-M. Tarascon, *J. Am. Chem. Soc.* **2011**, *133*, 16154.

[7] A. Baumann, X. Han, M. M. Butala, V. S. Thoi, *J. Am. Chem. Soc.* **2019**, *141*, 17891.

[8] H. Jiang, X.-C. Liu, Y. Wu, Y. Shu, X. Gong, F. Ke, H. Deng, *Angew. Chem. Int. Ed.* **2018**, *57*, 3916.

[9] P. Geng, L. Wang, M. Du, Y. Bai, W. Li, Y. Liu, S. Chen, P. Braunstein, Q. Xu, H. Pang, *Adv. Mater.* **2022**, *34*, 2107836.

[10] B. Y. Xia, Y. Yan, N. Li, H. Bin Wu, X. W. D. Lou, X. Wang, *Nat. Energy* **2016**, *1*, 15006.

[11] S. Hu, M. Yi, S. H. Siyal, D. Wu, H. Wang, Z. Zhu, J. Zhang, *J. Mater. Chem. A* **2021**, *9*, 15269.

[12] R. Wu, X. Qian, K. Zhou, J. Wei, J. Lou, P. M. Ajayan, *ACS Nano* **2014**, *8*, 6297.

[13] X. Chen, N. Wang, K. Shen, Y. Xie, Y. Tan, Y. Li, *ACS Appl. Mater. Interfaces* **2019**, *11*, 25976.

[14] Y. Cheng, X. Guo, Y. Xue, H. Pang, *Appl. Mater. Today* **2021**, *23*, 101048.

[15] J. Zhang, L. Wang, L. Xu, X. Ge, X. Zhao, M. Lai, Z. Liu, W. Chen, *Nanoscale* **2015**, *7*, 720.

[16] S. Cao, T. Chen, S. Zheng, Y. Bai, H. Pang, *Small Methods* **2021**, *5*, 2101070.

[17] G. Chen, Y. Li, W. Zhong, F. Zheng, J. Hu, X. Ji, W. Liu, C. Yang, Z. Lin, M. Liu, *Energy Storage Mater.* **2020**, *25*, 547.

[18] F. Zheng, D. Zhu, X. Shi, Q. Chen, *J. Mater. Chem. A* **2015**, *3*, 2815.
